# Quantifying human-animal contact rates in Malaysian Borneo: Influence of agricultural landscapes on contact with potential zoonotic disease reservoirs

**DOI:** 10.3389/fepid.2022.1057047

**Published:** 2023-01-18

**Authors:** Hannah Klim, Timothy William, Tock H. Chua, Giri S. Rajahram, Chris J. Drakeley, Miles W. Carroll, Kimberly M. Fornace

**Affiliations:** ^1^ Wellcome Centre for Human Genetics and Pandemic Sciences Institute, Nuffield Department of Medicine, University of Oxford, Oxford, United Kingdom; ^2^ Future of Humanity Institute, Faculty of Philosophy, University of Oxford, Oxford, United Kingdom; ^3^ Infectious Diseases Society Sabah-Menzies School of Health Research Clinical Research Unit, Kota Kinabalu, Malaysia; ^4^ Gleneagles Hospital, Kota Kinabalu, Malaysia; ^5^ Clinical Research Centre, Queen Elizabeth II Hospital, Kota Kinabalu, Malaysia; ^6^ Faculty of Medicine and Health Sciences, University of Malaysia Sabah, Kota Kinabalu, Malaysia; ^7^ Faculty of Infectious and Tropical Diseases, London School of Hygiene and Tropical Medicine, London, United Kingdom; ^8^ School of Biodiversity, One Health and Veterinary Medicine, University of Glasgow, Glasgow, United Kingdom; ^9^ Saw Swee Hock School of Public Health, National University of Singapore, Singapore, Singapore

**Keywords:** zoonoses, pathogen surveillance, spillover, climate change, land use - land cover change, reservoir species, human-animal interface

## Abstract

Changing landscapes across the globe, but particularly in Southeast Asia, are pushing humans and animals closer together and may increase the likelihood of zoonotic spillover events. Malaysian Borneo is hypothesized to be at high risk of spillover events due to proximity between reservoir species and humans caused by recent deforestation in the region. However, the relationship between landscape and human-animal contact rates has yet to be quantified. An environmentally stratified cross-sectional survey was conducted in Sabah, Malaysia in 2015, collecting geolocated questionnaire data on potential risk factors for contact with animals for 10,100 individuals. 51% of individuals reported contact with poultry, 46% with NHPs, 30% with bats, and 2% with swine. Generalised linear mixed models identified occupational and demographic factors associated with increased contact with these species, which varied when comparing wildlife to domesticated animals. Reported contact rates with each animal group were integrated with remote sensing-derived environmental data within a Bayesian framework to identify regions with high probabilities of contact with animal reservoirs. We have identified high spatial heterogeneity of contact with animals and clear associations between agricultural practices and high animal rates. This approach will help inform public health campaigns in at-risk populations and can improve pathogen surveillance efforts on Malaysian Borneo. This method can additionally serve as a framework for researchers looking to identify targets for future pathogen detection in a chosen region of study.

## Introduction

Global land changes caused by agricultural expansion and increased urbanization are widely thought to increase the risk of zoonotic spillover events (1–5). In particular, the reduction of primary forests has changed the density, species composition and distribution of disease reservoir hosts and will therefore influence the likelihood of interspecies transmission (2, 6, 7). This threat is critical in Southeast Asia, where deforestation rates are amongst the highest in the world (8, 9).

The island of Borneo has seen a particularly devastating amount of land change over the last 50 years, during which time an estimated 50% of the original forest area was lost (10, 11). These once forested regions are often being replaced with oil palm plantations (10, 11). This destruction of natural habitats will significantly impact the island's biodiversity over time, which is currently home to at least 10,000 different species of plants and 1,000 animals (12, 13). Not only is it ecologically essential to preserve these species and their habitats but, as forests are destroyed, it is hypothesized that some species will be forced closer to human populations, while others may go extinct. There are an estimated 245 species of forest vertebrates on Borneo, which have the potential to carry zoonotic pathogens with them if they move from once remote regions towards human settlements (10). These risks have been exemplified by emergence of the zoonotic simian malaria parasite *Plasmodium knowlesi* in this region, with human risks strongly associated with deforestation (14).

Malaysian Borneo is hypothesized to be at high risk of zoonotic spillover events precisely due to this proximity between reservoir species and humans caused by deforestation and the intensification of agriculture in the region (3, 15). A zoonotic spillover event occurs when a pathogen spreads from an animal into humans (16). These events can result in fatal outcomes partly due to the absence of prior immunity to a zoonotic pathogen. Over the last two decades alone, epidemics and pandemics caused by viral zoonotic pathogens have placed a burden on international healthcare systems and cost many lives (17–20).

These outbreaks but particularly the ongoing 2019 coronavirus disease (COVID-19) pandemic caused by SARS-CoV-2, have led many to call for the improvement of existing surveillance methods for viral zoonotic pathogens (21–26). While human-animal contact will not always lead to spillover events, it is a prerequisite for a spillover event to occur. It is therefore prudent for emerging infectious disease preparedness to study human-animal encounters. While assumptions can often be made about who is most likely to be in contact with reservoir species (farmers, hunters, etc.), these contact rates and risk factors for wildlife exposure have yet to be evaluated quantitatively. Several studies have highlighted the links between environmental changes, agricultural intensification, and spillover risk (1–5, 27), but there is still a need to spatially define this risk based on habitat preferences and human behaviours. Identifying populations and areas with high reported reservoir contact could help prioritise future surveillance efforts at the community level.

Here, we present a quantitative method for examining the risk of exposure to zoonotic pathogens in Malaysian Borneo, considering contact with known pathogen reservoir species, including swine, poultry, non-human primates, and bats. Swine and poultry have long been associated with a risk of influenza A virus zoonoses (28). Lethal, highly pathogenic H5 avian influenza virus was isolated from poultry in Sabah, Malaysian Borneo earlier this year (29), but no human infections have been reported thus far. Swine have also previously been associated with the transmission of Nipah virus in Peninsular Malaysia, a virus that causes a disease with a case fatality of between 40% and 75% (30). Fruit bats are another suspected reservoir for Nipah virus and may have contributed to previous spillover events, hence their inclusion in this study (31). Finally, non-human primates (NHPs) were also chosen for closer examination in this study due to the spread of Reston Ebolavirus in the nearby Philippines (32), as well as the transmission of simian zoonotic malaria in Sabah (33), which has previously been linked to high reported contact with macaques (14, 34). As these species are each well-known pathogen reservoirs, understanding human exposure to them is vital (28, 30, 35, 36).

In this study, we describe rates of human-animal contact within the region, define individual and demographic risk factors for contact, and develop predictive risk maps of potential zoonotic hotspots using environmental and land cover data. The high-risk individuals and regions identified through this analysis will be excellent candidates for future surveillance and serological studies.

## Methods

### Study population

An environmentally stratified, population-based cross-sectional survey was conducted in 2015 in Northern Sabah in Malaysian Borneo. The original purpose of this survey was to identify risk factors for malaria as described by Fornace et al. (3, 37). Briefly, a two-stage randomised sampling approach was used to survey 919 villages (clusters) from 4 districts in Northern Sabah, Malaysian Borneo, with an average population of 90 individuals and 36 households. Villages were classified into three groups (strata) based on the proportion of forest cover in 2014 within a 2 km radius of the centre of the village (14). To obtain 95% confidence and 80% power for estimating the seroprevalence of zoonotic malaria, it was calculated that a sample size of 883 households per strata would be required (2,650 in total) (3, 15). To meet this requirement, 20 households per selected village were randomly chosen. If the village had less than 20 households, then all households were included. This was supplemented with a random selection of other villages from the same strata until the target sample size was met (3).

**Figure 1:**
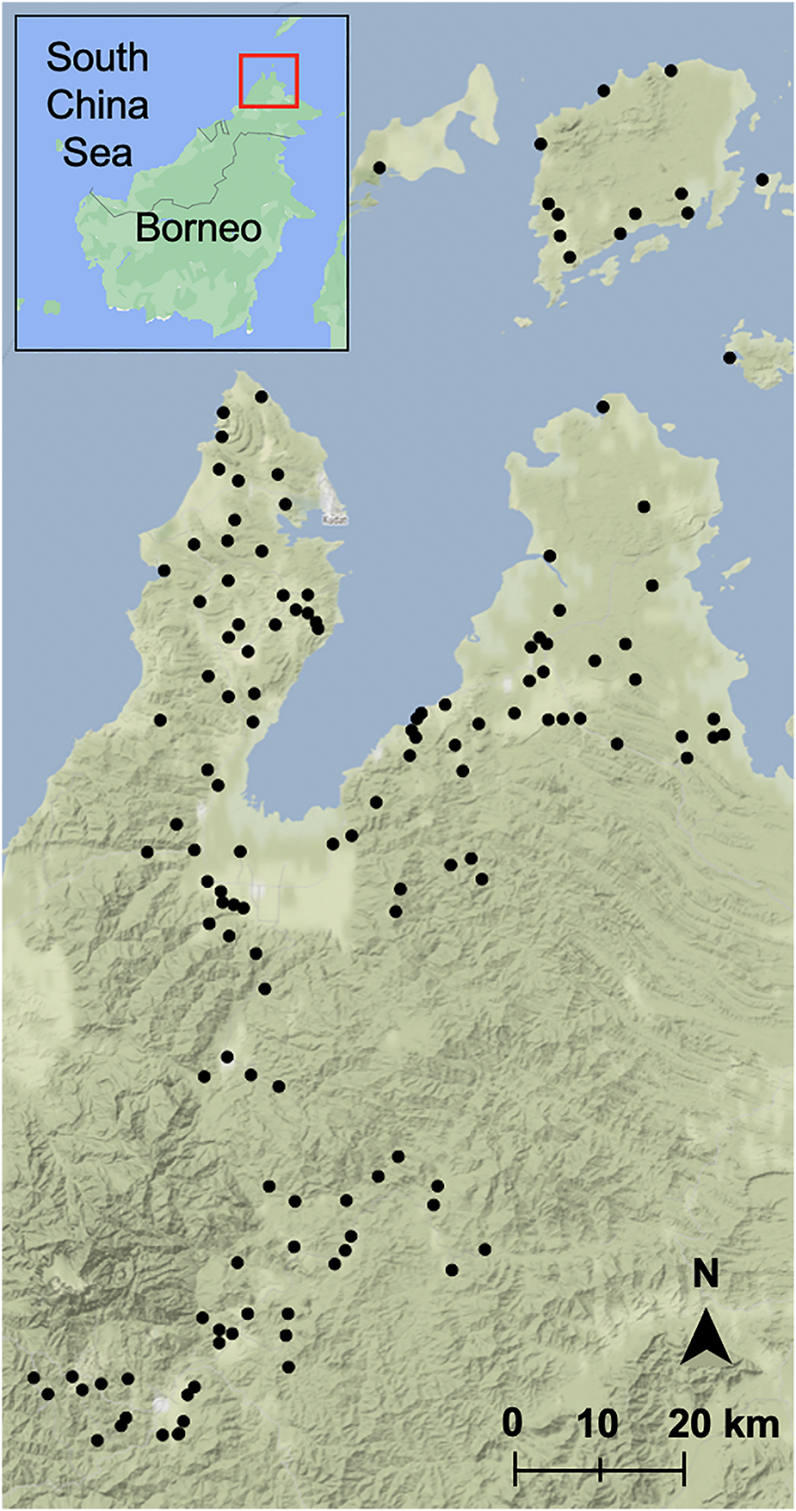
Sampled villages in Sabah, Malaysia. Map was built using village latitudes and longitudes with the ggmap package (39) in RStudio version 1.3.1093 (40, 41) with data from OpenStreetMap (61) and map tiles by Stamen Design (62). Inset map was built with Google Maps (60), red box indicates sampling region.

In total, 10,100 individuals from 2,650 households were asked to respond to a questionnaire including a variety of questions on demographic characteristics, habits, occupation, socioeconomic status, and animal contact (Figure 1). A household socioeconomic status index was created from survey data by Fornace et al. (3) based on education, assets, land, and construction. This socioeconomic index was divided into quartiles. Average travel times to nearby hospitals and clinics were also divided into quartiles based on community and patient interviews and modelled travel times (38). All individuals residing in the randomly selected households for the past month were asked to participate in the survey, excluding those younger than 3 months and those who could not be reached after three attempts. Survey responses from consenting individuals were collected with Pendragon Forms VI (Pendragon Software Corporation, Chicago, IL, USA).

### Ethical approval

Approval for this study was obtained from the Medical Research Sub-Committee of the Malaysian Ministry of Health (NMRR-14-713-21117) and the Research Ethics Committee of the London School of Hygiene and Tropical Medicine (8340). Written informed consent was obtained from all study participants.

### Rates of animal contact

Based on self-reported questionnaire data, individuals were classified as reporting contact with four potential reservoir species. For swine and poultry, ownership of these animals was considered a daily interaction. For non-human primates (NHPs), individuals were specifically asked if they saw these animals each day, week, month, or year. Whether individuals owned NHPs as pets or saw them around the house was included in the individual risk factor analysis. For bats, individuals were asked if they had seen a bat with no time frame associated.

For NHPs, the total number of individuals reporting daily sightings out of the total number of individuals sampled per village was calculated to map the proportion of each village's sample population interacting with NHPs. The same method was used for bats, but the individuals who responded yes were not assumed to have seen bats daily. For swine and poultry, daily sightings were replaced with ownership to calculate the proportion of each village interacting with these animals.

Maps displaying the proportion of each village interacting with a zoonotic reservoir species were built based on village latitudes and longitudes and coloured according to the proportions calculated from the survey responses using the ggmap package (39) in RStudio version 1.3.1093 (40, 41). The number of individuals sampled per village ranged from 5 to 107 across 170 villages (10,100 individuals in total).

### Individual risk factor analysis

Binomial generalized linear mixed models (GLMMs) were fit to understand individual risk factors which were associated with animal contact, as described in Fornace et al. (3) using the lme4 package (42) in R (40, 41). To adjust for the sampling design, household was included as a random effect, as well as a variety of fixed effects adapted from questionnaire responses. A univariate analysis was first used to select from 95 possible explanatory variables; those with *p* ≤ 0.2 were included in the development of a final model (Supplementary Tables S1–S4). Log likelihood ratio tests were then used to identify the final model, through a parsimonious forward stepwise approach with a logit link fit. Final adjusted odds ratios and 95% confidence intervals were calculated using the broom.mixed package (7) in RStudio version 1.3.1093 (40, 41) (Supplementary Tables S5–S8).

### Environmental risk factor analysis

We then aimed to assess the spatial distribution of human-animal contact to develop predictive risk maps. As demographic data was not available for all locations within the study site, we only considered environmental predictors. Binomial models were used to model contact with four categories of zoonotic reservoir species (NHPs, bats, swine, and poultry) with the outcome as the proportion of individuals per household reporting contact.

All households included in the study were geolocated and integrated with remote sensing-derived environmental data on land cover and climatic factors (37). Variable selection was conducted using generalized linear models (GLMs) with 21 of these potential environmental covariates. Data on elevation, aspect, and slope were obtained from the ASTER Digital Global Elevation Model (43) by Fornace et al. (3). The average annual normalised difference vegetation index (NDVI), which quantifies greenness of vegetation was calculated from Moderate Resolution Imaging Spectroradiometer 16-day composites at 250 m resolution (44) as in Fornace et al. (14). Precipitation seasonality, 1,970–2,000 (coefficient of variation), mean diurnal range, 1,970–2,000 (°C), minimum temperature of the coldest month, 1,970–2,000 (°C), average temperature, 1,970–2,000 (°C), precipitation of the wettest month. 1,970–2,000 (mm), maximum temperature of the warmest month, 1,970–2,000 (°C), and population density were extracted from the WorldClim dataset (45, 46). Household distance from mangroves, agricultural land, irrigated farmland, the sea, old forest, bush forest, roads, oil palm plantations, and rubber plantations were calculated by Fornace et al. (3).

The possible predictor variables were mean-centred and scaled. Univariate analysis was first used to identify variables with *p* ≤ 0.2 to consider in the development of final models (Supplementary Table S9). As mentioned above, the final variable selection used a parsimonious forward stepwise approach of log-likelihood ratio tests, with selected variables assessed for inclusion into geostatistical models (Supplementary Tables S10–S13).

For all multivariate models, spatial autocorrelation of the residuals was assessed with Moran's *I* with *p* < 0.05 considered statistically significant (Supplementary Table S14). For models demonstrating residual spatial autocorrelation, geostatistical models of animal contact were developed using a Bayesian framework with integrated nested Laplace approximations (INLA) using the R-INLA package (47, 48). Spatial effects were modelled as a Matérn covariance function, using the stochastic partial differential equation (SPDE) method (49). Spatial effects were included for all risk maps except those relating to swine, which did not have statistically significant spatial autocorrelation (Supplementary Figure S2). For the model intercepts and fixed effects coefficients, weakly informative priors of normal (0, 100) were used (50).

The final models were evaluated using the deviance information criteria (DIC) and area under the receiver operating curve (AUC). Posterior probabilities were estimated using 1,000 posterior samples. These posterior probabilities were then used to predict the probability of animal contact across the entire Sabah region. Uncertainty for these predictions was visualized through standard deviation (Supplementary Figure S1). Models with an AUC of less than 0.5 are not able to accurately predict the outcome beyond random chance, while values of 1 would indicate models that can predict outcomes perfectly (51). DIC was used to compare final models with their non-spatial counterparts.

Final maps were visualized with ggplot2 (52) in R using the viridis (53) colour palette.

## Results

### Distributions of animal contact

**Figure 2:**
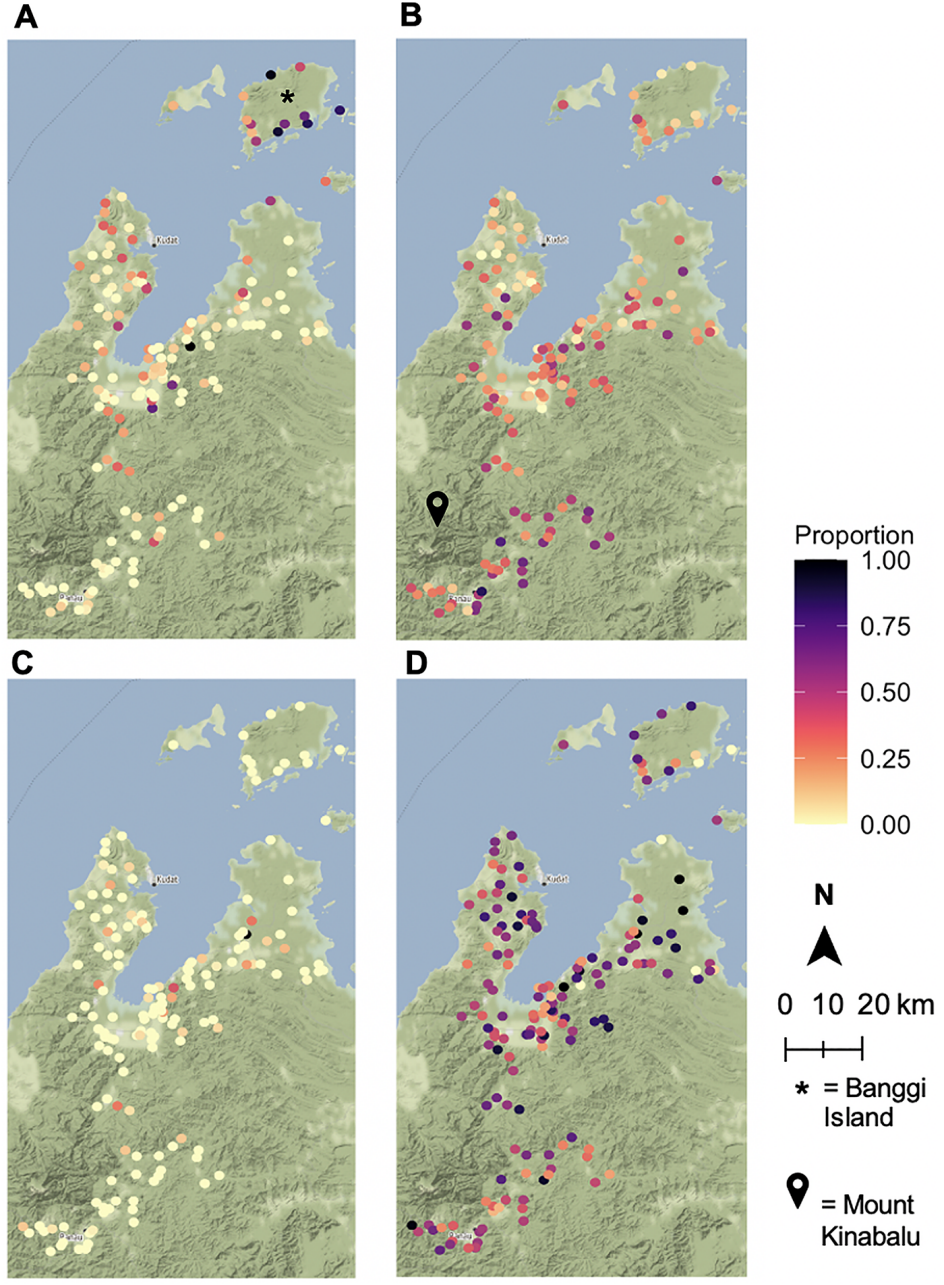
Human-animal contact rates by village. Proportion of sampled villagers interacting with (**A**) non-human primates, (**B**) bats, (**C**) swine, (**D**) poultry. Animal sightings and ownership were based on responses from a questionnaire. Maps were built using village latitudes and longitudes with the ggmap package (39) in RStudio version 1.3.1093 (40, 41) with data from OpenStreetMap (61) and map tiles by Stamen Design (62).

Maps displaying the proportion of a village's sampled population interacting with swine, poultry, bats, and non-human primates in Northern Sabah are displayed in Figure 2. 10,100 individuals from 2,650 households in 170 villages were included in this study. The sample population ranges in age from 3 months to 105 years, with a mean of 29 and a median of 25. The population was 47% men (*n* = 4,776) and 53% women (*n* = 5,324).

30% of individuals reported having seen a bat (*n* = 2,983). 50% of those who reported seeing a bat were male, and the group had a mean age of 32 with a median age of 30. Individuals in the study cohort were also asked about the frequency of NHP sightings with 617 individuals (6% of the population) reporting daily, 1,774 weekly (18%), 1,080 monthly (11%), and 1,180 annual (12%) NHP sightings. 5,449 individuals, or 54% of the population, reported no sightings of NHPs. The differences in age and gender distributions for these wild animal sightings were mostly minimal versus that of the overall population. For daily NHP sightings, the mean age was 32 and the median was 30, while the group was 48% male. For weekly sightings the mean and medians were the same, but the population was 53% male. Those who saw NHPs once a month had an average age of 33 with a median of 32 and were 51% male. The average age of those who never saw NHPs was 26, with a median of 19, and this group was 45% male. For annual NHP sightings, the group who responded yes had similar age distributions to the other groupings (mean = 31, median = 28), but this group was only 24% male.

Relative to the level of poultry ownership, swine ownership is much less prevalent in the villages sampled. Only 229 individuals in the study reported swine ownership, while 5,137 individuals reported poultry ownership. These differences can be attributed to religious practices that ban pork consumption.

There are clear geographic distributions of the wild animal reservoir species in this study. Particularly high clusters of bat sightings in villages located near Mount Kinabalu and Taman Negara Gunung Kinabalu, which is a large, forested nature preserve (Figure 2B). Overall, daily NHP sightings are heavily concentrated on Banggi Island, which is the island just north of mainland Sabah (Figure 2A). Due to the small sample size, there were few villages with a high proportion of swine owners (Figure 2C). The distribution of poultry ownership appears to be spread somewhat evenly throughout the region (Figure 2D).

### Individual risk factors with contact with animals

**Figure 3:**
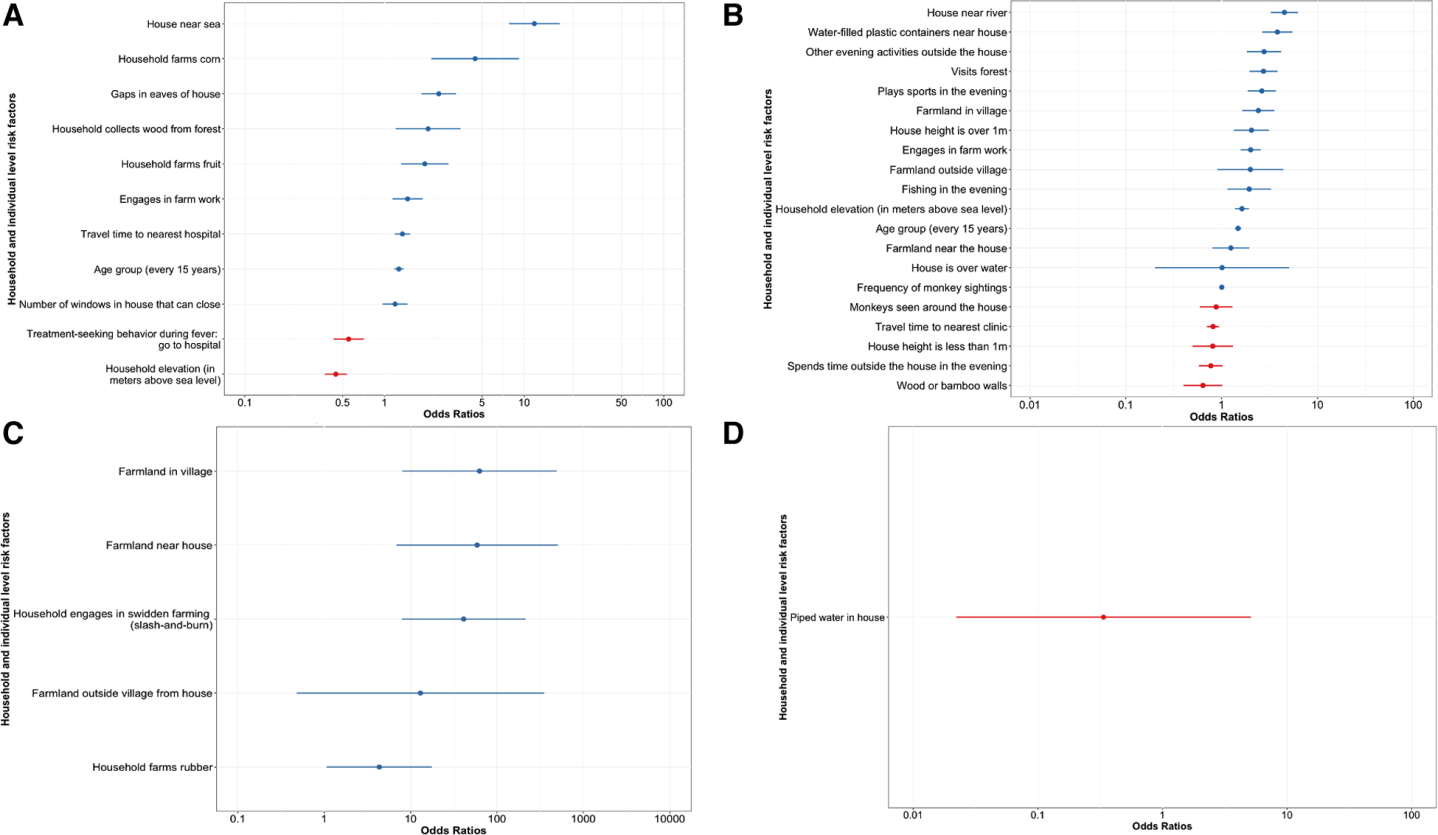
Adjusted odds ratios for fixed effects for household and individual level factors impact the odds of animal exposure. The combination of predictors which yielded the model with the highest log likelihood is shown with household as the random effect for (**A**) NHPs (−2157.854), (**B**) bats (−4094.4), (**C**) poultry (−2037.7), and (**D**) swine (−272.94). A univariate analysis was first used to select from 95 possible explanatory variables; those with *p* ≤ 0.2 were included in the development of a final model. This model was made with the lme4 package (42) and analysed with the broom.mixed package (7) in RStudio version 1.3.1093 (40, 41).

Exposure to zoonotic reservoir species (bats, swine, poultry, or NHPs) was modelled based on survey responses. The mixed effects modelling results are presented in Figure 3, which displays adjusted odds ratios and 95% confidence intervals for both individual and household level fixed effects (numeric values are provided in the Supplementary Information). For wildlife animals, bats and NHPs, age and farm work were associated with increased odds of animal sightings (Figures 3A,B). Increased sightings of bats were additionally associated with certain evening activities, forest visits, proximity to rivers, and household elevation, amongst other factors (Figure 3B). The odds of increased NHP sightings were positively associated with corn farming, fruit farming, collecting wood from the forest, living near the sea, and having windows that can close (Figure 3A). Additionally, populations with the highest contact with NHPs had the lowest probability of seeking treatment when they had a fever. Being male was statistically significant in the univariate analysis for both NHPs and bats, but not in the final model (Supplementary Tables S1, S2).

For poultry and swine, only household-level effects showed statistical significance, as all individuals in a household were in contact with the same domesticated animals. With respect to poultry, farmland near the house, swidden farming, and rubber farming were all associated with increased odds of poultry ownership (Figure 3C). In the initial univariate analysis for poultry ownership, many of the significant factors were related to household farming practices and livestock ownership (Supplementary Table S3).

Piped water inside the house was the only significant factor in the swine multivariate analysis (Figure 3D). Having piped water inside one's house was associated with decreased odds of swine ownership. The household collecting wood from the forest, house height, ethnicity, treatment-seeking behaviour, and insect screens in houses were all significant in the univariate analysis, but not in the multivariate final model (Supplementary Table S4).

### Predictive risk mapping

**Figure 4:**
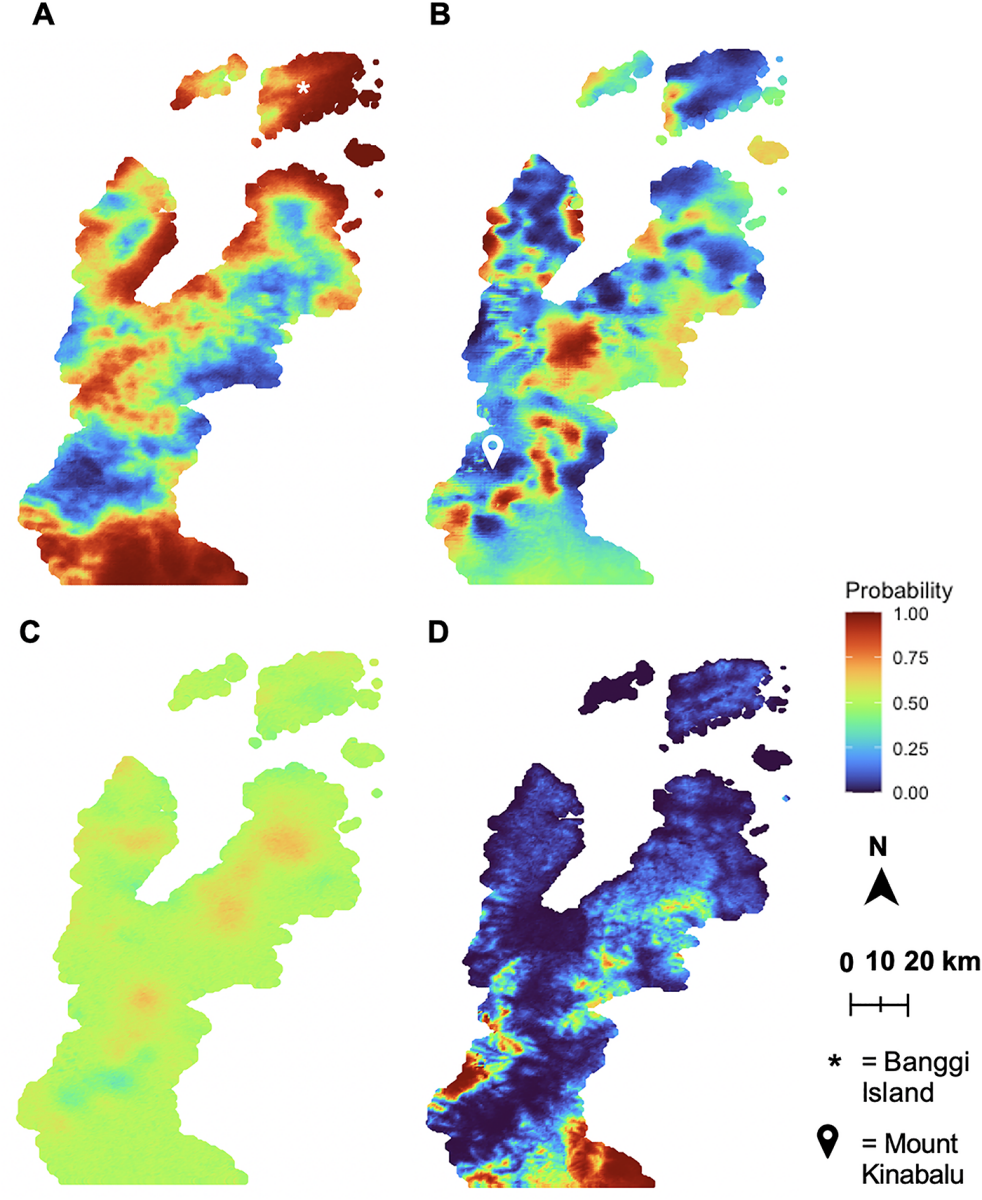
Environmental and land use factors may model high-risk regions for probability of exposure to potential zoonotic reservoir species. Spatial patterns of mean posterior estimated probability of animal exposure were modelled using R-INLA (47, 48) and the SPDE method (49) as the spatial effect. The final models were evaluated using the DIC and AUC. (**A**) is the model for NHPs with a DIC of 33087.32 and AUC of 0.7591; (**B**) is the model for bats, where the DIC = 29957.03 and AUC = 0.9595; (**C**) models the estimated probability of poultry exposure and DIC = 60218.65 and AUC = 0.5009; (**D**) is the model for swine exposure, with a DIC of 8288.93 and AUC of 0.7817. Spatial effects were included for all risk maps except (**D**) swine, which did not have statistically significant spatial autocorrelation through Moran's I. For the model intercepts and fixed effects coefficients, weakly informative priors of Normal (0, 100) were used (50).

Environmental and land cover data was incorporated with survey responses to generate spatial models of the Northern Sabah region. These maps display the mean posterior estimated probability of animal exposure based on geostatistical modelling (Figure 4). Models of contact with NHPs have a moderate predictive power (AUC = 0.7591), while models of bat contact have high predictive power (AUC = 0.9595). The model of swine contacts also had moderate predictive power, although the spatial effect was not included (AUC = 0.7817). The poultry contact model showed poor predictive power with an AUC = 0.5009. Except for the swine model, all models with a spatial effect had a lower DIC than their non-spatial counterparts.

Regions with a high predicted probability of NHP sightings are seen throughout the map, but particularly near the coastline and on Banggi Island (Figure 4A). Regions with a low predicted probability of NHP sightings tended to be the internal regions of the map, including the villages around Mount Kinabalu and the surrounding forested nature preserve. The opposite effect was observed for bats, which had higher predicted probabilities of sightings in these internal regions away from the coast and near Mount Kinabalu (Figure 4B). There were fewer bat sightings hotspots than of NHP sightings, which is expected based on the survey responses.

Pockets of higher predicted probability of poultry exposure are centred around village clusters, rather than specific geographic features (Figure 4C). In the map of exposure to swine, the inclusion of the spatial effect did not improve model fit. There are a few regions with a high estimated probability of swine exposure (Figure 4D), but the uncertainty around these predictions is high (Supplementary Figure S1).

## Discussion

This study has presented a novel approach to identifying high-risk populations for zoonotic disease surveillance in Sabah. Since animal contact is a prerequisite for a spillover event (5), it is crucial to understand populations and risk factors associated with contact with these species, especially those which are not domesticated. The results have shown that there is a high level of individual and spatial heterogeneity underscoring animal contact on Malaysian Borneo, which should be considered by surveillance programmes when targeting screening. Indeed, the risk factors and high-risk regions enumerated through this analysis can be used to identify priorities for surveillance and vaccination within this region. The populations identified through this analysis challenge standard assumptions, in that animal contact was not defined by predicted high-risk occupations, such as hunting. Instead, animal contact was driven by proximity to agriculture and natural habitats of wildlife species.

Identification of risk factors by species allows us to consider the threat of specific infectious agents and the differences between domesticated and wild animals. For example, on Banggi Island and many of the coastal Sabah regions, we noted that the predicted probability of NHP exposure is at its highest (Figure 4A). These areas would therefore be priorities for monitoring of a variety of infectious agents including Simian retroviruses (54), herpesvirus B (55), or Reston ebolavirus (32). NHP exposure has previously been reported as a risk factor for zoonotic malaria within this region (3, 33, 34, 38), so understanding where this contact occurs will be critical to malaria prevention efforts.

Conversely, the predicted probability of bat exposure is highest in Sabah's non-coastal and higher elevation regions of Sabah, including nearly the thickly forested Mount Kinabalu (Figure 4B). Indeed, from the survey data, visiting forests was positively associated with the odds of seeing a bat (Figure 3). Bats are associated with the transmission a variety of viruses to humans, including SARS-CoV-1 (35), Ebola viruses (56), and Marburg virus (57). The opposing regions for bat versus NHP sightings were also validated through the survey-based risk factor analysis; the odds of a bat sighting were decreased by sighting NHPs around the house and those who saw NHPs less frequently had increased odds of seeing a bat (Figure 3). The differences between contact with NHPs and bats are likely driven by habitat preferences and species distribution, which have been captured in part by the questionnaire data. These results highlight the risk of disturbing natural habitats and increasing agriculture at human-animal interfaces.

Certain demographic and socioeconomic factors were identified as important for increasing the odds of sighting a wildlife species, but not for exposure to a domesticated species. Those over 55 years of age had the highest odds of interacting with a wildlife species, along with those living in houses located near bodies of water (river/sea) and houses in remote locations (Quartile 4 of distance to the nearest hospital or clinic) (Figures 3A,B). Engaging in farming was a key risk factor in animal exposure, for both domesticated and wildlife species. These results are consistent with previous studies highlighting risk factors for emerging zoonotic disease in this region, which included age, male sex, activities in the forest, farm work, and proximity to oil palms and farmland (3, 15, 34, 58). These results imply that while occupational and behavioural factors drive contact rates, environmental covariates can serve as a proxy for modelling animal contact when other information is not available.

The risk maps presented here additionally highlight that the odds of sighting particular animals increase with proximity to farmland. Proximity to oil palm plantations, rubber farms, and irrigated farmland were reoccurring risk factors (Supplementary Information). The high deforestation rates and replacement of old forest with farmland on Malaysian Borneo mean that the proximity between households and farmland will only increase (10, 11). This could increase the risk of exposure to wild animals, and therefore, exposure to zoonotic disease. Although farm work and visiting a forest are hardly practices which can be stopped, these results seek to bring awareness to the quantifiable risk from these activities. The results presented here, along with the aforementioned studies, highlight the threat of oil palm plantations which destroy natural wildlife habitats. This practice of mass deforestation and replacement with industrial farmland can and should be slowed to mitigate the risk of emerging infectious disease events on Borneo.

While the geostatistical maps predict exposure patterns for wild animals (bats and NHPs), a limitation of this approach is that environmental covariates do not appear to be well-suited to predicting exposure to domesticated species. This pattern can be seen through the AUC values: the poultry model AUC indicated worse predictive ability than the AUC results for NHPs and bats, which both had moderate predictive ability. For swine, the model was not spatially correlated, which is likely due to the small number of swine owners within the survey population (roughly 2%). As swine is domesticated, it is predicted that the risk maps would follow a similar pattern to that of poultry—with exposure clusters around villages—if there were more swine owners in the region. Additionally, the risk factor analysis for swine revealed that piped water was the only factor which was associated (negatively) with the odds of swine ownership. However, piped water is more likely to be available to Muslim households due to government development action. As Islamic beliefs prohibit rearing, contact, and consumption, Muslims do not keep swine. Thus, it is more likely to be religion, than piped water, that has the association with swine ownership (59).

Another limitation of this study is that the survey responses about animal contact are self-reported and therefore, subject to bias. However, this bias in the data is mitigated to an extent through the large number of participants from a variety of demographic backgrounds. Furthermore, the specific species of bats and NHPs sighted by study participants are unknown. Species specificity is vital for certain pathogens, such as zoonotic malaria (33) and Nipah virus (31). More generally, animal contact, as reported here, is a term that lacks detail regarding the nature of these interactions. In future surveys, it could improve the models shown here to understand the type of contact, such as close contact versus mere proximity, eating bushmeat, or guano collection. This study did not record sources of indirect animal contact from vectors. Future studies could extend this work by integrating the models presented here with a deeper analysis of contact and through comparisons to vector distribution.

Thus far, maps outlining the threat of zoonotic transfer have tended to be on a global scale (2, 4). World maps generated by Allen et al. and Carlson & Albery et al. estimate Borneo and the surrounding area as a region which is likely to experience zoonotic spillover events due to bat encounters (4) and biodiversity (2), along with a variety of climatic and demographic factors. By contrast, the work presented here provides a similar output, but on a smaller and more detailed scale, which can be implemented immediately within Sabah. This study allows public health officials to pinpoint villages or coordinates which are most at-risk and is specific to Sabah's unique demographic and environmental topography. In settings where resources need to be prioritized, the methods presented here could be used as a novel tool for targeted monitoring of spillover events from wild animals. To our knowledge, an approach integrating such detailed population and environmental data to build risk maps has not previously been presented. A serological analysis of the at-risk individuals identified through this study will be an important next step in assessing the predictive power of our models. This method could be applied to other regions where cross-sectional surveys have been conducted if spatial information is available. Future studies illustrating detailed risk maps for other regions will provide valuable insights into the widespread impacts of climate change on zoonotic risk.

## Data Availability

The data analyzed in this study is subject to the following licenses/restrictions: All code and environmental data are available on reasonable request. As survey data includes identifiable information on household coordinates, data is only available following approval by the relevant ethics committees in Malaysia and the UK. Requests to access these datasets should be directed to kimberly.fornace@glasgow.ac.uk. Example code for all models is provided at https://github.com/hklim06/Quantifying-human-animal-contact-rates-in-Malaysian-Borneo.
