# Supplementary material for: Quantifying human-animal contact rates in Malaysian Borneo: Influence of agricultural landscapes on contact with potential zoonotic disease reservoirs
Source: Front Epidemiol. 2023 Jan 18;2:1057047. doi: 10.3389/fepid.2022.1057047 (PMC10910987; doi:10.3389/fepid.2022.1057047)
Supplement: Supplementary file 1 [file Datasheet1.docx]

Supplementary Material

# Univariate risk factor analysis

| ***Variable*** |  | **Total number** | **Number of NHP owners** | **Crude odds ratios (95% CI)** | **P value** |
| --- | --- | --- | --- | --- | --- |
| *Monkeys seen around the house* | | |  |  |  |
|  | No | 8816 | 2619 | Ref |  |
|  | Yes | 1284 | 364 | 8.38e+60 (1.62e+58 - 4.33e+63) | < 0.001 |
| *Frequency of monkey sightings* | | |  |  |  |
|  | Never | 5449 | 1275 | Ref |  |
|  | Daily | 1180 | 373 | 3.29e+158 (3.29e+158 - 3.29e+158) | |
|  | Monthly | 1774 | 628 | 162754.79 (162754.79 - 162754.79) | |
|  | Weekly | 1080 | 485 | 3.83e+22 (3.83e+22 - 3.83e+22) | |
|  | Yearly | 617 | 222 | 2.72 (2.72 - 2.72) | < 0.001 |
| *Contact with monkeys* | |  |  |  |  |
|  | No | 5478 | 1285 | Ref |  |
|  | Yes | 4622 | 1698 | 3.23e+30 (7.99e+28 - 1.31e+32) | < 0.001 |
| *Contact with long-tailed macaques* | | |  |  |  |
|  | No | 5478 | 1285 | Ref |  |
|  | Yes | 4622 | 1698 | 3.23e+30 (7.99e+28 - 1.31e+32) | < 0.001 |
| *Monkeys seen around the village* | | |  |  |  |
|  | No | 7433 | 2006 | Ref |  |
|  | Yes | 2667 | 977 | 1.36e+21 (1.97622e+19 - 9.39e+22) | < 0.001 |
| *Sea near the house* | |  |  |  |  |
|  | No | 9262 | 2771 | Ref |  |
|  | Yes | 838 | 212 | 8.58e+51 (1.05e+46 - 6.98e+57) | < 0.001 |
| *Travel time to nearest hospital* | | |  |  |  |
|  | Quartile 1 | 2467 | 713 | Ref |  |
|  | Quartile 2 | 2599 | 678 | 879477.01 (12.37 - 62534479576) | |
|  | Quartile 3 | 2533 | 900 | 26.87 (0 - 2310173.03) |  |
|  | Quartile 4 | 2501 | 692 | 1.54e+28 (2.04e+23 - 1.16e+33) | < 0.001 |
| *Elevation (in metres above sea level)* | | |  |  |  |
|  | Under 50 | 5701 | 1321 | Ref |  |
|  | 50-250 | 2248 | 814 | 0 (0 - 0) |  |
|  | 250-500 | 1227 | 565 | 0 (0 - 0) |  |
|  | Over 500 | 924 | 283 | 0 (0 - 0) |  |
| *Occupation* |  |  |  |  |  |
|  | Farmer | 655 | 103 | Ref |  |
|  | Fishing | 30 | 6 | 1.81322e+17 (1.47224e+12 - 2.23e+22) | |
|  | None | 3100 | 222 | 0 (0 - 0) |  |
|  | Office/shop | 56 | 8 | 0 (0 - 0) |  |
|  | Other | 138 | 13 | 55 (0.01 - 415944.48) |  |
|  | Palm oil plantation | 38 | 8 | 309048.44 (0.11 - 8.52951e+11) | |
|  | Rubber plantation | 126 | 46 | 1.39 (0 - 39369.19) |  |
|  | Student | 1747 | 184 | 0 (0 - 0) | < 0.001 |
| *Household has pet monkey* | | |  |  |  |
|  | No | 9863 | 2898 | Ref |  |
|  | Yes | 237 | 85 | 4.04e+69 (1.05e+58 - 1.55e+81) | < 0.001 |
| *House height* | |  |  |  |  |
|  | Ground level | 3478 | 959 | Ref |  |
|  | Less than 1m | 2094 | 562 | 1721289.55 (28.1 - 1.05428e+11) | |
|  | Over 1m | 4416 | 1442 | 6.70251e+11 (78264805.18 - 5.73996e+15) | |
|  | Over water | 112 | 20 | 8.93e+80 (2.98e+65 - 2.67e+96) | < 0.001 |
| *Age* |  |  |  |  |  |
|  | Under 5 | 937 | 38 | Ref |  |
|  | 5-15 | 1652 | 151 | 197163.61 (2063.88 - 18835134.02) | |
|  | 15-30 | 1164 | 138 | 29758696.44 (237370.39 - 3730793877) | |
|  | 30-55 | 1458 | 197 | 8857694510 (95650541.26 - 8.20265e+11) | |
|  | Over 55 | 855 | 93 | 69567604122 (250641985 - 1.9309e+13) | < 0.001 |
| *Ethnicity* |  |  |  |  |  |
|  | Bajau | 319 | 46 | Ref |  |
|  | Dusun | 3628 | 343 | 0 (0 - 0) |  |
|  | Other | 496 | 45 | 4.68 (0 - 411346.48) |  |
|  | Rungus | 1384 | 159 | 0 (0 - 0) |  |
|  | Sungoi | 239 | 24 | 0 (0 - 0) | < 0.001 |
| *Socioeconomic status* | |  |  |  |  |
|  | Quartile 1 | 2251 | 690 | Ref |  |
|  | Quartile 2 | 2526 | 805 | 0 (0 - 0) |  |
|  | Quartile 3 | 2626 | 759 | 0 (0 - 0) |  |
|  | Quartile 4 | 2697 | 729 | 0 (0 - 0) | < 0.001 |
| *Piped water inside the house* | | |  |  |  |
|  | No | 4296 | 1222 | Ref |  |
|  | Yes | 5804 | 1761 | 0 (0 - 0) | < 0.001 |
| *Gaps in eaves of the house* | | |  |  |  |
|  | No | 5561 | 1690 | Ref |  |
|  | Yes | 4539 | 1293 | 6.84734e+16 (2.36099e+13 - 1.99e+20) | < 0.001 |
| *Monkeys raid household crops* | | |  |  |  |
|  | No | 9006 | 2577 | Ref |  |
|  | Yes | 1094 | 406 | 2.65e+26 (6.47e+20 - 1.09e+32) | < 0.001 |
| *Household has pet monkey* | | |  |  |  |
|  | No | 8267 | 2326 | Ref |  |
|  | Yes | 1833 | 657 | 4.82007e+18 (1.46242e+14 - 1.59e+23) | < 0.001 |
| *House has a toilet* | |  |  |  |  |
|  | No | 1279 | 276 | Ref |  |
|  | Yes | 8821 | 2707 | 0 (0 - 0) | < 0.001 |
| *Household has cattle* | |  |  |  |  |
|  | No | 9907 | 2947 | Ref |  |
|  | Yes | 193 | 36 | 1.88e+49 (4.92e+36 - 7.2e+61) | < 0.001 |
| *Household farms corn* | |  |  |  |  |
|  | No | 9854 | 2900 | Ref |  |
|  | Yes | 246 | 83 | 2.89e+41 (4.68e+30 - 1.79e+52) | < 0.001 |
| *Individual engages in farm work* | | |  |  |  |
|  | No | 5082 | 430 | Ref |  |
|  | Yes | 984 | 187 | 1238616.26 (31088.54 - 49348413.95) | < 0.001 |
| *Collect wood in forest* | |  |  |  |  |
|  | No | 5997 | 595 | Ref |  |
|  | Yes | 69 | 22 | 5.096e+14 (36437310071 - 7.1271e+18) | < 0.001 |
| *Cleared land in the past year* | | |  |  |  |
|  | No | 5997 | 595 | Ref |  |
|  | Yes | 69 | 22 | 5.096e+14 (36437310071 - 7.1271e+18) | < 0.001 |
| *Involved in construction in the past year* | | | |  |  |
|  | No | 5997 | 595 | Ref |  |
|  | Yes | 69 | 22 | 5.096e+14 (36437310071 - 7.1271e+18) | < 0.001 |
| *Other activities in evenings* | | |  |  |  |
|  | Fishing | 65 | 13 | Ref |  |
|  | None | 4667 | 372 | 0 (0 - 0) |  |
|  | Other | 198 | 20 | 0 (0 - 0) |  |
|  | Visiting outside house | 818 | 134 | 0 (0 - 0.31) |  |
|  | Sport | 318 | 78 | 0 (0 - 0) | < 0.001 |
| *Usually bathe outside* | |  |  |  |  |
|  | No | 7510 | 2091 | Ref |  |
|  | Yes | 2590 | 892 | 308150052.6 (1062911.01 - 89336222485) | < 0.001 |
| *Use smoke to prevent mosquitoes* | | |  |  |  |
|  | No | 9242 | 2678 | Ref |  |
|  | Yes | 858 | 305 | 1.97012e+12 (495832266.1 - 7.82802e+15) | < 0.001 |
| *Go to forest* |  |  |  |  |  |
|  | No | 5798 | 548 | Ref |  |
|  | Yes | 268 | 69 | 114162271 (418903.19 - 31112257981) | < 0.001 |
| *Walk to work or school through forest* | | |  |  |  |
|  | No | 5661 | 531 | Ref |  |
|  | Yes | 405 | 86 | 12775400.24 (83696.99 - 1950020581) | < 0.001 |
| *River near the house* | |  |  |  |  |
|  | No | 4911 | 1009 | Ref |  |
|  | Yes | 5189 | 1974 | 0 (0 - 0) | < 0.001 |
| *Floors in house made of concrete or tile* | | | |  |  |
|  | No | 6854 | 1964 | Ref |  |
|  | Yes | 3246 | 1019 | 0 (0 - 0) | < 0.001 |
| *Well observed near house* | |  |  |  |  |
|  | No | 8435 | 2309 | Ref |  |
|  | Yes | 1665 | 674 | 1.14809e+12 (21650625.59 - 6.08806e+16) | < 0.001 |
| *Pond near house* | |  |  |  |  |
|  | No | 8157 | 2086 | Ref |  |
|  | Yes | 1943 | 897 | 0 (0 - 0) | < 0.001 |
| *Distance to nearest clinic* | |  |  |  |  |
|  | Quartile 1 | 2495 | 698 | Ref |  |
|  | Quartile 2 | 2592 | 862 | 2111.82 (0.02 - 181221290.8) | |
|  | Quartile 3 | 2420 | 675 | 46.21 (0 - 5087109.53) |  |
|  | Quartile 4 | 2593 | 748 | 1.4095e+12 (17570020.1 - 1.13072e+17) | < 0.001 |
| *Usually walk to work* | |  |  |  |  |
|  | No | 4688 | 418 | Ref |  |
|  | Yes | 1378 | 199 | 2504.24 (91.3 - 68686.07) | < 0.001 |
| *Household farms rubber* | |  |  |  |  |
|  | No | 6564 | 1760 | Ref |  |
|  | Yes | 3536 | 1223 | 0 (0 - 0) | < 0.001 |
| *Treatment- seeking behaviour during fever: obtain medcines from clinic* | | | | |  |
|  | No | 4490 | 368 | Ref |  |
|  | Yes | 1576 | 249 | 96251.69 (439.18 - 21094969.48) | < 0.001 |
| *Household uses insecticides for farming* | | | |  |  |
|  | No | 7162 | 1968 | Ref |  |
|  | Yes | 2938 | 1015 | 0 (0 - 0) | < 0.001 |
| *Travel to/or from work or school between 11pm-6am* | | | | |  |
|  | No | 5583 | 485 | Ref |  |
|  | Yes | 483 | 132 | 86502.54 (332.46 - 22506813.52) | < 0.001 |
| *Self-reported previous malaria diagnosis* | | | |  |  |
|  | No | 5382 | 506 | Ref |  |
|  | Yes | 684 | 111 | 2922.22 (41.37 - 206404.49) | < 0.001 |
| *Travel to/from work or school between 5pm and 10pm* | | | | |  |
|  | No | 5153 | 513 | Ref |  |
|  | Yes | 913 | 104 | 1573 (26.43 - 93619.14) | < 0.001 |
| *Go to forest at night (5pm-6am)* | | |  |  |  |
|  | No | 5891 | 563 | Ref |  |
|  | Yes | 175 | 54 | 83308.11 (85.51 - 81164824.92) | 0.001 |
| *Distance of farming land from the house* | | | |  |  |
|  | Near the house | 2110 | 573 | Ref |  |
|  | Same village | 3810 | 1480 | 0 (0 - 5993.28) | 0.001 |
|  | No farmland |  |  |  |  |
|  | Outside the village | 3747 | 773 | 257 (0 - 14737000.58) |  |
| *Toilet is inside house* | |  |  |  |  |
|  | No | 5787 | 1856 | Ref |  |
|  | Yes | 4313 | 1127 | 0 (0 - 0.01) | 0.001 |
| *Go to forest at night (5pm-10pm)* | | |  |  |  |
|  | No | 5908 | 573 | Ref |  |
|  | Yes | 158 | 44 | 75671.61 (55.38 - 103389848.1) | 0.002 |
| *Sleep under a bednet* | |  |  |  |  |
|  | No | 2170 | 587 | Ref |  |
|  | Yes | 7930 | 2396 | 4687.23 (16.3 - 1348009.04) | 0.003 |
| *Occupation place* | |  |  |  |  |
|  | In district | 1140 | 136 | Ref |  |
|  | Around the house | 3429 | 280 | 0.09 (0 - 4.57) |  |
|  | In village | 1437 | 191 | 56.65 (0.59 - 5422.36) |  |
|  | Different district | 60 | 10 | 0.78 (0 - 845529.05) | 0.003 |
| *Household has livestock* | |  |  |  |  |
|  | No | 10082 | 2979 | Ref |  |
|  | Yes | 18 | 4 | 6.11e+53 (3.57287e+17 - 1.05e+90) | 0.004 |
| *Wood or bamboo walls* | |  |  |  |  |
|  | No | 1957 | 534 | Ref |  |
|  | Yes | 8143 | 2449 | 3138547.84 (118.35 - 83232495624) | 0.004 |
| *Household owns a buffalo* | | |  |  |  |
|  | No | 9985 | 2944 | Ref |  |
|  | Yes | 115 | 39 | 2.94e+23 (21228722.93 - 4.07e+39) | 0.004 |
| *Where are stays outside of the village* | | |  |  |  |
|  | None | 9686 | 2830 | Ref |  |
|  | District | 241 | 85 | 13534.41 (0.35 - 523550029) | |
|  | Forest | 17 | 8 | 2133.52 (0 - 4.59321e+17) | |
|  | Outside | 145 | 54 | 4165.42 (0.01 - 2283554412) | |
|  | Plantation | 11 | 6 | 2.75e+36 (1.4383e+14 - 5.27e+58) | 0.005 |
| *Age of house* | |  |  |  |  |
|  | 1 to 5 years | 1704 | 418 | Ref |  |
|  | Over 5 years | 7993 | 2448 | 0 (0 - 0) |  |
|  | Under 1 year | 368 | 108 | 0 (0 - 0.03) |  |
|  | Unknown | 35 | 9 | 0 (0 - 364.43) | 0.006 |
| *Household farms palm oil* | | |  |  |  |
|  | No | 9106 | 2673 | Ref |  |
|  | Yes | 994 | 310 | 0 (0 - 0) | 0.007 |
| *Amount of land farmed* | |  |  |  |  |
|  | No farmland | 3800 | 792 | Ref |  |
|  | Quartile 1 | 428 | 132 | 0 (0 - 2.18) |  |
|  | Quartile 2 | 3895 | 1303 | 0 (0 - 0.01) |  |
|  | Quartile 3 | 1977 | 756 | 0 (0 - 0.27) | 0.009 |
| *Kitchen outside house* | |  |  |  |  |
|  | No | 9661 | 2825 | Ref |  |
|  | Yes | 439 | 158 | 91249935290 (396.89 - 2.09797e+19) | 0.01 |
| *Have stayed outside the village in the past month* | | | |  |  |
|  | No | 9678 | 2827 | Ref |  |
|  | Yes | 422 | 156 | 32457.63 (10.03 - 105015691.3) | 0.012 |
| *Use a fan to prevent mosquitoes* | | |  |  |  |
|  | No | 8124 | 2295 | Ref |  |
|  | Yes | 1976 | 688 | 2551.26 (5.08 - 1281185.96) | 0.014 |
| *Gender* |  |  |  |  |  |
|  | No | 3326 | 328 | Ref |  |
|  | Yes | 2740 | 289 | 24.45 (1.9 - 314.28) | 0.014 |
| *Lake near house* | |  |  |  |  |
|  | No | 10018 | 2960 | Ref |  |
|  | Yes | 82 | 23 | 1.31e+23 (10115.09 - 1.7e+42) | 0.018 |
| *Hunting in forest* | |  |  |  |  |
|  | No | 5998 | 594 | Ref |  |
|  | Yes | 68 | 23 | 98481.1 (4.78 - 2028157888) | 0.023 |
| *Treatment- seeking behaviour during fever: go to hospital* | | | | |  |
|  | No | 2931 | 199 | Ref |  |
|  | Yes | 3135 | 418 | 0 (0 - 0.5) | 0.026 |
| *Household owns a goat* | |  |  |  |  |
|  | No | 10011 | 2963 | Ref |  |
|  | Yes | 89 | 20 | 5.00942e+18 (12.05 - 2.08e+36) | 0.038 |
| *Treatment- seeking behaviour during fever sees traditional healer* | | | | |  |
|  | No | 6060 | 616 | Ref |  |
|  | Yes | 6 | 1 | 1.12787e+16 (1.27 - 1e+32) | 0.049 |
| *Length of time resident at current house* | | |  |  |  |
|  | 1 to 5 years |  | 409 | Ref |  |
|  | Over 5 years | | 2455 | 0 (0 - 0.05) |  |
|  | Less than 1 year | | 113 | 0 (0 - 2007.71) |  |
|  | Unknown |  | 6 | 0 (0 - 11316671389) | 0.057 |
| *Household farms fruit* | |  |  |  |  |
|  | No | 8527 | 2258 | Ref |  |
|  | Yes | 1573 | 725 | 21683.36 (0.24 - 1971233791) | 0.087 |
| *Go to forest at night (11pm-6am)* | | |  |  |  |
|  | No | 6042 | 604 | Ref |  |
|  | Yes | 24 | 13 | 334532.04 (0.11 - 1.05412e+12) | 0.096 |
| *Swidden farming* | |  |  |  |  |
|  | No | 5338 | 1246 | Ref |  |
|  | Yes | 4762 | 1737 | 0 (0 - 4.43) | 0.109 |
| *Collect wood in forest* | |  |  |  |  |
|  | No | 7716 | 2196 | Ref |  |
|  | Yes | 2384 | 787 | 0 (0 - 4.43) | 0.109 |
| *Windows in the house can close* | | |  |  |  |
|  | None | 1088 | 284 | Ref |  |
|  | Some | 4546 | 1560 | 356.62 (0 - 220597709.2) | |
|  | All | 4466 | 1139 | 0.09 (0 - 59130.01) | 0.157 |
| *River near the house* | |  |  |  |  |
|  | No | 9565 | 2752 | Ref |  |
|  | Yes | 535 | 231 | 2386.87 (0.05 - 119172483.1) | 0.159 |
| *Treatment- seeking behaviour during fever: don't seek treatment* | | | | |  |
|  | No | 5161 | 541 | Ref |  |
|  | Yes | 905 | 76 | 66.14 (0.07 - 62426.58) | 0.23 |
| *Household farms vegetables* | | |  |  |  |
|  | No | 8454 | 2315 | Ref |  |
|  | Yes | 1646 | 668 | 269.31 (0 - 16762605.53) | 0.32 |
| *Treatment- seeking behaviour during fever: take traditional medicines* | | | | |  |
|  | No | 5905 | 582 | Ref |  |
|  | Yes | 161 | 35 | 123.72 (0.01 - 2754209.76) | 0.346 |
| *Insect screens in house* | |  |  |  |  |
|  | No | 9410 | 2725 | Ref |  |
|  | Yes | 690 | 258 | 0 (0 - 6744) | 0.38 |
| *House collects food from forest* | |  |  |  |  |
|  | No | 8074 | 2258 | Ref |  |
|  | Yes | 2026 | 725 | 0.02 (0 - 414.58) | 0.422 |
| *Corrugated iron roof* | |  |  |  |  |
|  | No | 1136 | 351 | Ref |  |
|  | Yes | 8964 | 2632 | 165.5 (0 - 79263549.13) | 0.444 |
| *Don’t use any mosquito prevention* | | |  |  |  |
|  | No | 9852 | 2892 | Ref |  |
|  | Yes | 248 | 91 | 0.01 (0 - 2827.23) | 0.445 |
| *Household collects medicine from the forest* | | | |  |  |
|  | No | 8627 | 2442 | Ref |  |
|  | Yes | 1473 | 541 | 0.01 (0 - 1471.94) | 0.457 |
| *Have slept outside walls (out of houses) in the past month* | | | | |  |
|  | No | 10050 | 2959 | Ref |  |
|  | Yes | 50 | 24 | 579.49 (0 - 1.78263e+11) | 0.523 |
| *Household has rice paddies* | | |  |  |  |
|  | No | 8791 | 2483 | Ref |  |
|  | Yes | 1309 | 500 | 0.02 (0 - 5074.89) | 0.553 |
| *Use window screens to prevent mosquitoes* | | | |  |  |
|  | No | 10044 | 2967 | Ref |  |
|  | Yes | 56 | 16 | 412.21 (0 - 2.19077e+11) | 0.557 |
| *Report taking anti-malaria medication* | | |  |  |  |
|  | No | 6025 | 606 | Ref |  |
|  | Yes | 41 | 11 | 13.65 (0 - 435771104.9) | 0.767 |

**Supplemental Table 1. Crude odds ratios for fixed effects for household and individual level factors impact the odds of NHP exposure.** Variables with p$\leq$0.2 were included in the development of a final model. This model was made with the lme4 package*(1)* and analysed with the broom.mixed package(2) in RStudio version 1.3.1093*(3, 4)*.

| ***Variable*** |  | **Total number** | **Number of bat owners** | **Crude odds ratios (95% CI)** | **P value** |
| --- | --- | --- | --- | --- | --- |
| *Frequency of monkey sightings* | | |  |  |  |
|  | Never | 5449 | 1275 | Ref |  |
|  | Daily | 1180 | 373 | 11.41 (7.23 - 18.01) | |
|  | Monthly | 1774 | 628 | 9.47 (6.94 - 12.92) | |
|  | Weekly | 1080 | 485 | 14.28 (9.88 - 20.64) | |
|  | Yearly | 617 | 222 | 6.5 (4.3 - 9.83) | < 0.001 |
| *Contact with monkeys* | |  |  |  |  |
|  | No | 5478 | 1285 | Ref |  |
|  | Yes | 4622 | 1698 | 9.5 (7.34 - 12.29) | < 0.001 |
| *Contact with long-tailed macaques* | | |  |  |  |
|  | No | 5478 | 1285 | Ref |  |
|  | Yes | 4622 | 1698 | 9.5 (7.34 - 12.29) | < 0.001 |
| *Age* |  |  |  |  |  |
|  | Under 5 | 1160 | 176 | Ref |  |
|  | 5-15 | 2611 | 691 | 5.53 (3.98 - 7.7) | |
|  | 15-30 | 1944 | 607 | 9.59 (6.79 - 13.55) | |
|  | 30-55 | 2833 | 1001 | 15.52 (11.06 - 21.78) | |
|  | Over 55 | 1552 | 508 | 13.81 (9.34 - 20.43) | < 0.001 |
| *Individual engages in farmwork* | | |  |  |  |
|  | No | 8075 | 2069 | Ref |  |
|  | Yes | 2025 | 914 | 5.55 (4.47 - 6.88) | < 0.001 |
| *Occupation* |  |  |  |  |  |
|  | Farmer | 1412 | 602 | Ref |  |
|  | Fishing | 180 | 55 | 0.68 (0.34 - 1.37) | |
|  | None | 4751 | 1180 | 0.22 (0.17 - 0.28) | |
|  | Office/shop | 371 | 132 | 0.69 (0.43 - 1.1) | |
|  | Other | 296 | 95 | 0.62 (0.37 - 1.02) | |
|  | Palm oil plantation | 96 | 34 | 0.89 (0.39 - 2.04) | |
|  | Rubber plantation | 254 | 117 | 2.04 (1.17 - 3.57) | |
|  | Student | 2740 | 768 | 0.28 (0.21 - 0.36) | < 0.001 |
| *Go to forest* |  |  |  |  |  |
|  | No | 9345 | 2593 | Ref |  |
|  | Yes | 755 | 390 | 9.41 (6.81 - 13.02) | < 0.001 |
| *River near the house* | |  |  |  |  |
|  | No | 4911 | 1009 | Ref |  |
|  | Yes | 5189 | 1974 | 7.83 (5.56 - 11.03) | < 0.001 |
| *Self-reported previous malaria diagnosis* | | | |  |  |
|  | No | 8771 | 2380 | Ref |  |
|  | Yes | 1329 | 603 | 4.04 (3.19 - 5.12) | < 0.001 |
| *Monkeys seen around the village* | | |  |  |  |
|  | No | 7433 | 2006 | Ref |  |
|  | Yes | 2667 | 977 | 4.01 (3.14 - 5.12) | < 0.001 |
| *Distance of farming land from the house* | | |  |  |  |
|  | Near the house | 2110 | 573 | Ref |  |
|  | Same village | 3810 | 1480 | 4.11 (2.62 - 6.43) | |
|  | No farmland | 3747 | 773 | 0.44 (0.28 - 0.69) | |
|  | Outside the village | 433 | 157 | 3.02 (1.29 - 7.06) | < 0.001 |
| *Plastic containers of water seen near the house* | | | |  |  |
|  | No | 7473 | 1798 | Ref |  |
|  | Yes | 2627 | 1185 | 8.26 (5.69 - 12.01) | < 0.001 |
| *Pond near house* | |  |  |  |  |
|  | No | 8157 | 2086 | Ref |  |
|  | Yes | 1943 | 897 | 9.82 (6.5 - 14.86) | < 0.001 |
| *Go to forest at night (5pm-6am)* | | |  |  |  |
|  | No | 9622 | 2737 | Ref |  |
|  | Yes | 478 | 246 | 7.41 (5.09 - 10.78) | < 0.001 |
| *Elevation (in metres above sea level)* | | |  |  |  |
|  | Under 50 | 5701 | 1321 | Ref |  |
|  | 50-250 | 2248 | 814 | 5.15 (3.42 - 7.74) | |
|  | 250-500 | 1227 | 565 | 12.4 (7.36 - 20.91) | |
|  | Over 500 | 924 | 283 | 2.86 (1.63 - 5.04) | < 0.001 |
| *Other activities in evenings* | | |  |  |  |
|  | Fishing | 263 | 111 | Ref |  |
|  | None | 7332 | 2000 | 0.21 (0.13 - 0.36) | |
|  | Other | 491 | 235 | 1.07 (0.57 - 2.01) | |
|  | Visiting outside house | 1403 | 375 | 0.21 (0.12 - 0.37) | |
|  | Sport | 611 | 262 | 0.63 (0.35 - 1.13) | < 0.001 |
| *Go to forest at night (5pm-10pm)* | | |  |  |  |
|  | No | 9664 | 2762 | Ref |  |
|  | Yes | 436 | 221 | 6.4 (4.34 - 9.45) | < 0.001 |
| *Amount of land farmed* | |  |  |  |  |
|  | No farmland | 3800 | 792 | Ref |  |
|  | Quartile 1 | 428 | 132 | 3.16 (1.4 - 7.13) | |
|  | Quartile 2 | 3895 | 1303 | 5.25 (3.56 - 7.74) | |
|  | Quartile 3 | 1977 | 756 | 7.05 (4.42 - 11.23) | < 0.001 |
| *Swidden farming* | |  |  |  |  |
|  | No | 5338 | 1246 | Ref |  |
|  | Yes | 4762 | 1737 | 4.85 (3.46 - 6.81) | < 0.001 |
| *Occupation place* | |  |  |  |  |
|  | In district | 1864 | 556 | Ref |  |
|  | Around the house | 5538 | 1421 | 0.64 (0.63 - 0.64) | |
|  | In village | 2586 | 971 | 1.55 (1.55 - 1.55) | |
|  | Different district | 112 | 35 | 1.14 (1.14 - 1.15) | < 0.001 |
| *Hunting in forest* | |  |  |  |  |
|  | No | 9900 | 2866 | Ref |  |
|  | Yes | 200 | 117 | 8.98 (5.22 - 15.47) | < 0.001 |
| *Uncovered water containers seen around the house* | | | |  |  |
|  | Unknown | 209 | 43 | Ref |  |
|  | No | 3518 | 731 | 0.92 (0.29 - 2.88) | |
|  | Yes | 6373 | 2209 | 4.14 (1.34 - 12.8) | < 0.001 |
| *Household farms fruit* | |  |  |  |  |
|  | No | 8527 | 2258 | Ref |  |
|  | Yes | 1573 | 725 | 6.34 (4.04 - 9.97) | < 0.001 |
| *Usually walk to work* | |  |  |  |  |
|  | No | 7490 | 2014 | Ref |  |
|  | Yes | 2610 | 969 | 2.05 (1.71 - 2.45) | < 0.001 |
| *Use insecticide* | |  |  |  |  |
|  | No | 5455 | 1394 | Ref |  |
|  | Yes | 4645 | 1589 | 2.46 (1.95 - 3.12) | < 0.001 |
| *Travel to/from work or school between 11pm and 6am* | | | | |  |
|  | No | 10016 | 2932 | Ref |  |
|  | Yes | 84 | 51 | 20.5 (8.62 - 48.73) | < 0.001 |
| *Ethnicity* |  |  |  |  |  |
|  | Bajau | 884 | 203 | Ref |  |
|  | Dusun | 5074 | 1799 | 3.02 (1.81 - 5.02) | |
|  | Other | 1050 | 191 | 0.77 (0.42 - 1.4) | |
|  | Rungus | 2682 | 653 | 0.94 (0.54 - 1.63) | |
|  | Sungoi | 410 | 137 | 2.73 (1.21 - 6.16) | < 0.001 |
| *Collect wood in forest* | |  |  |  |  |
|  | No | 9870 | 2872 | Ref |  |
|  | Yes | 230 | 111 | 6.75 (4.03 - 11.3) | < 0.001 |
| *Cleared land in the past year* | | |  |  |  |
|  | No | 9870 | 2872 | Ref |  |
|  | Yes | 230 | 111 | 6.75 (4.03 - 11.3) | < 0.001 |
| *Involved in construction in the past year* | | | |  |  |
|  | No | 9870 | 2872 | Ref |  |
|  | Yes | 230 | 111 | 6.75 (4.03 - 11.3) | < 0.001 |
| *Walk to work or school through forest* | | |  |  |  |
|  | No | 9146 | 2583 | Ref |  |
|  | Yes | 954 | 400 | 2.77 (2.11 - 3.64) | < 0.001 |
| *Well observed near house* | |  |  |  |  |
|  | No | 8435 | 2309 | Ref |  |
|  | Yes | 1665 | 674 | 4.61 (2.97 - 7.17) | < 0.001 |
| *Household farms vegetables* | | |  |  |  |
|  | No | 8454 | 2315 | Ref |  |
|  | Yes | 1646 | 668 | 4.29 (2.76 - 6.68) | < 0.001 |
| *Report taking anti-malaria medication* | | |  |  |  |
|  | No | 10026 | 2930 | Ref |  |
|  | Yes | 74 | 53 | 19.01 (6.82 - 52.99) | < 0.001 |
| *Usually bathe outside* | |  |  |  |  |
|  | No | 7510 | 2091 | Ref |  |
|  | Yes | 2590 | 892 | 2.34 (1.78 - 3.08) | < 0.001 |
| *Household uses insecticides for farming* | | | |  |  |
|  | No | 7162 | 1968 | Ref |  |
|  | Yes | 2938 | 1015 | 3.1 (2.14 - 4.48) | < 0.001 |
| *Gender* |  |  |  |  |  |
|  | No | 5324 | 1479 | Ref |  |
|  | Yes | 4776 | 1504 | 1.52 (1.32 - 1.76) | < 0.001 |
| *Treatment- seeking behaviour during fever: don't seek treatment* | | | | |  |
|  | No | 8775 | 2703 | Ref |  |
|  | Yes | 1325 | 280 | 0.38 (0.27 - 0.55) | < 0.001 |
| *Household collects food from the forest* | | |  |  |  |
|  | No | 8074 | 2258 | Ref |  |
|  | Yes | 2026 | 725 | 2.94 (1.95 - 4.44) | < 0.001 |
| *Household has rice paddies* | | |  |  |  |
|  | No | 8791 | 2483 | Ref |  |
|  | Yes | 1309 | 500 | 3.58 (2.18 - 5.87) | < 0.001 |
| *Household collects medicine from the forest* | | | |  |  |
|  | No | 8627 | 2442 | Ref |  |
|  | Yes | 1473 | 541 | 3.28 (2.05 - 5.25) | < 0.001 |
| *Household farms rubber* | |  |  |  |  |
|  | No | 6564 | 1760 | Ref |  |
|  | Yes | 3536 | 1223 | 2.43 (1.71 - 3.45) | < 0.001 |
| *Travel to/or from work or school between 5pm and 10pm* | | | | |  |
|  | No | 9211 | 2676 | Ref |  |
|  | Yes | 889 | 307 | 1.78 (1.41 - 2.23) | < 0.001 |
| *Windows in the house can close* | | |  |  |  |
|  | None | 1088 | 284 | Ref |  |
|  | Some | 4546 | 1560 | 2.09 (1.21 - 3.62) | |
|  | All | 4466 | 1139 | 0.86 (0.49 - 1.49) | < 0.001 |
| *Toilet is inside house* | |  |  |  |  |
|  | No | 5787 | 1856 | Ref |  |
|  | Yes | 4313 | 1127 | 0.44 (0.32 - 0.62) | < 0.001 |
| *Usually bathe at river* | |  |  |  |  |
|  | No | 9565 | 2752 | Ref |  |
|  | Yes | 535 | 231 | 3.39 (2.03 - 5.67) | < 0.001 |
| *House has a toilet* | |  |  |  |  |
|  | No | 1279 | 276 | Ref |  |
|  | Yes | 8821 | 2707 | 3.19 (1.93 - 5.29) | < 0.001 |
| *House height* | |  |  |  |  |
|  | Ground level | 3478 | 959 | Ref |  |
|  | Less than 1m | 2094 | 562 | 0.85 (0.53 - 1.36) | |
|  | Over 1m | 4416 | 1442 | 2.04 (1.39 - 2.98) | |
|  | Over water | 112 | 20 | 0.2 (0.04 - 1.09) | < 0.001 |
| *Household has pet monkey* | | |  |  |  |
|  | No | 8267 | 2326 | Ref |  |
|  | Yes | 1833 | 657 | 2.37 (1.55 - 3.63) | < 0.001 |
| *Use a fan to prevent mosquitoes* | | |  |  |  |
|  | No | 8124 | 2295 | Ref |  |
|  | Yes | 1976 | 688 | 1.82 (1.35 - 2.46) | < 0.001 |
| *Travel to/from work or school between 11pm and 6am* | | | | |  |
|  | No | 9211 | 2676 | Ref |  |
|  | Yes | 889 | 307 | 1.83 (1.35 - 2.48) | < 0.001 |
| *Monkeys raid household crops* | | |  |  |  |
|  | No | 9006 | 2577 | Ref |  |
|  | Yes | 1094 | 406 | 2.73 (1.61 - 4.62) | < 0.001 |
| *Sea near the house* | |  |  |  |  |
|  | No | 9262 | 2771 | Ref |  |
|  | Yes | 838 | 212 | 0.32 (0.17 - 0.59) | < 0.001 |
| *Collect wood in forest* | |  |  |  |  |
|  | No | 7716 | 2196 | Ref |  |
|  | Yes | 2384 | 787 | 2.05 (1.39 - 3.02) | < 0.001 |
| *Travel time to nearest hospital* | | |  |  |  |
|  | Quartile 1 | 2467 | 713 | Ref |  |
|  | Quartile 2 | 2599 | 678 | 0.86 (0.53 - 1.37) | |
|  | Quartile 3 | 2533 | 900 | 1.96 (1.22 - 3.16) | |
|  | Quartile 4 | 2501 | 692 | 0.77 (0.48 - 1.24) | < 0.001 |
| *Insect screens in house* | |  |  |  |  |
|  | No | 9410 | 2725 | Ref |  |
|  | Yes | 690 | 258 | 3.02 (3.02 - 3.03) | 0.001 |
| *Length of time resident at current house* | | | |  |  |
|  | 1 to 5 years | 1635 | 409 | Ref |  |
|  | Over 5 years | 8059 | 2455 | 2.48 (1.56 - 3.92) | |
|  | Less than 1 year | 385 | 113 | 1.67 (0.67 - 4.17) | |
|  | Unknown | 21 | 6 | 2.55 (0.11 - 57.57) | 0.001 |
| *Age of house* | |  |  |  |  |
|  | 1 to 5 years | 1704 | 418 | Ref |  |
|  | Over 5 years | 7993 | 2448 | 2.39 (1.52 - 3.74) | |
|  | Under 1 year | 368 | 108 | 1.75 (0.69 - 4.47) | |
|  | Unknown | 35 | 9 | 0.87 (0.06 - 12.88) | 0.002 |
| *Treatment- seeking behaviour during fever: go to hospital* | | | | |  |
|  | No | 4820 | 1414 | Ref |  |
|  | Yes | 5280 | 1569 | 1.43 (1.12 - 1.83) | 0.004 |
| *Treatment- seeking behaviour during fever: obtain medcines from clinic* | | | | | |
|  | No | 7085 | 2069 | Ref |  |
|  | Yes | 3015 | 914 | 1.4 (1.08 - 1.81) | 0.012 |
| *Use smoke to prevent mosquitoes* | | |  |  |  |
|  | No | 9242 | 2678 | Ref |  |
|  | Yes | 858 | 305 | 1.65 (1.11 - 2.44) | 0.013 |
| *Gaps in eaves of the house* | | |  |  |  |
|  | No | 5561 | 1690 | Ref |  |
|  | Yes | 4539 | 1293 | 0.66 (0.47 - 0.92) | 0.014 |
| *Kitchen outside house* | |  |  |  |  |
|  | No | 9661 | 2825 | Ref |  |
|  | Yes | 439 | 158 | 2.57 (1.18 - 5.61) | 0.018 |
| *Have slept outside walls (out of houses) in the past month* | | | | |  |
|  | No | 10050 | 2959 | Ref |  |
|  | Yes | 50 | 24 | 3.08 (1.04 - 9.09) | 0.04 |
| *Treatment- seeking behaviour during fever: take traditional medicines* | | | | | |
|  | No | 9672 | 2884 | Ref |  |
|  | Yes | 428 | 99 | 0.59 (0.34 - 1) | 0.047 |
| *Piped water inside the house* | | |  |  |  |
|  | No | 4296 | 1222 | Ref |  |
|  | Yes | 5804 | 1761 | 1.37 (0.98 - 1.92) | 0.065 |
| *Distance to nearest clinic* | |  |  |  |  |
|  | Quartile 1 | 2495 | 698 | Ref |  |
|  | Quartile 2 | 2592 | 862 | 1.7 (1.06 - 2.72) | |
|  | Quartile 3 | 2420 | 675 | 0.98 (0.6 - 1.59) | |
|  | Quartile 4 | 2593 | 748 | 1.12 (0.71 - 1.79) | 0.073 |
| *Household has cattle* | |  |  |  |  |
|  | No | 9907 | 2947 | Ref |  |
|  | Yes | 193 | 36 | 0.36 (0.1 - 1.24) | 0.103 |
| *Household farms palm oil* | | |  |  |  |
|  | No | 9106 | 2673 | Ref |  |
|  | Yes | 994 | 310 | 1.6 (0.91 - 2.82) | 0.105 |
| *Socioeconomic status* | |  |  |  |  |
|  | Quartile 1 | 2251 | 690 | Ref |  |
|  | Quartile 2 | 2526 | 805 | 1.26 (0.79 - 2.02) | |
|  | Quartile 3 | 2626 | 759 | 0.8 (0.5 - 1.28) | |
|  | Quartile 4 | 2697 | 729 | 0.74 (0.46 - 1.19) | 0.108 |
| *Sleep under a bednet* | |  |  |  |  |
|  | No | 2170 | 587 | Ref |  |
|  | Yes | 7930 | 2396 | 1.25 (0.94 - 1.66) | 0.125 |
| *Monkeys seen around the house* | | |  |  |  |
|  | No | 8816 | 2619 | Ref |  |
|  | Yes | 1284 | 364 | 1.32 (0.92 - 1.88) | 0.129 |
| *Household has pet monkey* | | |  |  |  |
|  | No | 9863 | 2898 | Ref |  |
|  | Yes | 237 | 85 | 2.36 (0.78 - 7.17) | 0.129 |
| *Wood or bamboo walls* | |  |  |  |  |
|  | No | 1957 | 534 | Ref |  |
|  | Yes | 8143 | 2449 | 1.37 (0.89 - 2.08) | 0.148 |
| *Household farms corn* | |  |  |  |  |
|  | No | 9854 | 2900 | Ref |  |
|  | Yes | 246 | 83 | 2.1 (0.76 - 5.81) | 0.155 |
| *Treatment- seeking behaviour during fever sees traditional healer* | | | | |  |
|  | No | 10086 | 2982 | Ref |  |
|  | Yes | 14 | 1 | 0.11 (0 - 4.1) | 0.155 |
| *Floors in house made of concrete or tile* | | | |  |  |
|  | No | 6854 | 1964 | Ref |  |
|  | Yes | 3246 | 1019 | 1.26 (0.88 - 1.81) | 0.202 |
| *Where are stays outside of the village* | | |  |  |  |
|  | None | 9686 | 2830 | Ref |  |
|  | District | 241 | 85 | 0.94 (0.56 - 1.58) | |
|  | Forest | 17 | 8 | 2.99 (0.62 - 14.47) | |
|  | Outside | 145 | 54 | 1.17 (0.59 - 2.33) | |
|  | Plantation | 11 | 6 | 11.14 (0.58 - 215.45) | 0.281 |
| *Swine owner* | |  |  |  |  |
|  | No | 9871 | 2912 | Ref |  |
|  | Yes | 229 | 71 | 1.73 (0.59 - 5.07) | 0.319 |
| *Use window screens to prevent mosquitoes* | | | |  |  |
|  | No | 10044 | 2967 | Ref |  |
|  | Yes | 56 | 16 | 1.54 (0.55 - 4.27) | 0.41 |
| *Household has livestock* | |  |  |  |  |
|  | No | 10082 | 2979 | Ref |  |
|  | Yes | 18 | 4 | 0.22 (0.01 - 9.17) | 0.412 |
| *Household owns a goat* | |  |  |  |  |
|  | No | 10011 | 2963 | Ref |  |
|  | Yes | 89 | 20 | 0.54 (0.1 - 3.01) | 0.481 |
| *Have stayed outside the village in the past month* | | | |  |  |
|  | No | 9678 | 2827 | Ref |  |
|  | Yes | 422 | 156 | 1.14 (0.76 - 1.71) | 0.517 |
| *Don’t use any mosquito prevention* | | |  |  |  |
|  | No | 9852 | 2892 | Ref |  |
|  | Yes | 248 | 91 | 1.08 (0.58 - 2.02) | 0.812 |
| *Lake near house* | |  |  |  |  |
|  | No | 10018 | 2960 | Ref |  |
|  | Yes | 82 | 23 | 1.16 (0.2 - 6.83) | 0.873 |
| *Corrugated iron roof* | |  |  |  |  |
|  | No | 1136 | 351 | Ref |  |
|  | Yes | 8964 | 2632 | 0.97 (0.57 - 1.66) | 0.922 |
| *Household owns a buffalo* | |  |  |  |  |
|  | No | 9985 | 2944 | Ref |  |
|  | Yes | 115 | 39 | 0.82 (0.82 - 0.82) | 1 |

**Supplemental Table 2. Crude odds ratios for fixed effects for household and individual level factors impact the odds of bat exposure.** Variables with p$\leq$0.2 were included in the development of a final model. This model was made with the lme4 package*(1)* and analysed with the broom.mixed package(2) in RStudio version 1.3.1093*(3, 4)*.

| ***Variable*** |  | **Total number** | **Number of poultry owners** | **Crude odds ratios (95% CI)** | **P value** |
| --- | --- | --- | --- | --- | --- |
| *Distance of farming land from the house* | | |  |  |  |
|  | Near the house | 2110 | 1307 | Ref |  |
|  | Same village | 3810 | 2336 | 1.15 (0.28-4.81) |  |
|  | No farmland | 3747 | 1262 | 0.00 (0.00-0.00) |  |
|  | Outside the village | 433 | 232 | 0.35 (0.02-7.14) | < 0.001 |
| *Amount of land farmed* | |  |  |  |  |
|  | No farmland | 3800 | 1284 | Ref |  |
|  | Quartile 1 | 428 | 268 | 2790.97 (215.25-36205.79) |  |
|  | Quartile 2 | 3895 | 2360 | 1019.53 (268.83-3866.47) |  |
|  | Quartile 3 | 1977 | 1225 | 1125.48 (230.82-5487.98) | < 0.001 |
| *Swidden farming* | |  |  |  |  |
|  | No | 5338 | 2093 | Ref |  |
|  | Yes | 4762 | 3044 | 761.74 (247.12-2348.05) | < 0.001 |
| *Household collects wood from forest* | |  |  |  |  |
|  | No | 7716 | 3453 | Ref |  |
|  | Yes | 2384 | 1684 | 458.02 (128.22-1636.13) | < 0.001 |
| *Household farms rubber* | |  |  |  |  |
|  | No | 6564 | 2904 | Ref |  |
|  | Yes | 3536 | 2233 | 106.28 (33.36-338.64) | < 0.001 |
| *Socioeconomic status* | |  |  |  |  |
|  | Quartile 1 | 2251 | 1374 | Ref |  |
|  | Quartile 2 | 2526 | 1494 | 0.74 (0.17-3.33) |  |
|  | Quartile 3 | 2626 | 1277 | 0.03 (0.01-0.15) |  |
|  | Quartile 4 | 2697 | 992 | 0.00 (0.00-0.02) | < 0.001 |
| *House height* | |  |  |  |  |
|  | Ground level | 3478 | 1548 | Ref |  |
|  | Less than 1m | 2094 | 1000 | 1.45 (0.31-6.86) |  |
|  | Over 1m | 4416 | 2583 | 20.72 (5.69-75.45) |  |
|  | Over water | 112 | 6 | 0.00 (0.00-0.00) | < 0.001 |
| *Toilet is inside house* | |  |  |  |  |
|  | No | 5787 | 3375 | Ref |  |
|  | Yes | 4313 | 1762 | 0.01 (0.00-0.05) | < 0.001 |
| *Household collects medicine from the forest* | | |  |  |  |
|  | No | 8627 | 4070 | Ref |  |
|  | Yes | 1473 | 1067 | 207.62 (51.71-833.67) | < 0.001 |
| *Household uses insecticides for farming* | |  |  |  |  |
|  | No | 7162 | 3270 | Ref |  |
|  | Yes | 2938 | 1867 | 86.48 (26.07-286.86) | < 0.001 |
| *Household collects food from the forest* | |  |  |  |  |
|  | No | 8074 | 3782 | Ref |  |
|  | Yes | 2026 | 1355 | 116.06 (32.33-416.63) | < 0.001 |
| *Monkeys raid household crops* | |  |  |  |  |
|  | No | 9006 | 4367 | Ref |  |
|  | Yes | 1094 | 770 | 83.39 (18.48-376.28) | < 0.001 |
| *Household has rice paddies* | |  |  |  |  |
|  | No | 8791 | 4287 | Ref |  |
|  | Yes | 1309 | 850 | 39.63 (8.84-177.57) | < 0.001 |
| *Household farms vegetables* | |  |  |  |  |
|  | No | 8454 | 4147 | Ref |  |
|  | Yes | 1646 | 990 | 24.63 (5.91-102.7) | < 0.001 |
| *Piped water inside the house* | |  |  |  |  |
|  | No | 4296 | 2449 | Ref |  |
|  | Yes | 5804 | 2688 | 0.09 (0.03-0.28) | < 0.001 |
| *Household farms fruit* | |  |  |  |  |
|  | No | 8527 | 4170 | Ref |  |
|  | Yes | 1573 | 967 | 20.02 (4.51-88.14) | < 0.001 |
| *Sea near the house* | |  |  |  |  |
|  | No | 9262 | 4836 | Ref |  |
|  | Yes | 838 | 301 | 0.04 (0.01-0.18) | < 0.001 |
| *Distance to nearest clinic* | |  |  |  |  |
|  | Quartile 1 | 2495 | 1077 | Ref |  |
|  | Quartile 2 | 2592 | 1259 | 2.17 (0.46-10.20) |  |
|  | Quartile 3 | 2420 | 1323 | 18.68 (3.73-93.42) |  |
|  | Quartile 4 | 2593 | 1478 | 17.1 (3.58-81.71) | < 0.001 |
| *Household has livestock* | |  |  |  |  |
|  | No | 10082 | 5119 | Ref |  |
|  | Yes | 18 | 18 | 4.39E+24 (0-Inf) | 0.001 |
| *River near the house* | |  |  |  |  |
|  | No | 4911 | 2332 | Ref |  |
|  | Yes | 5189 | 2805 | 6.50 (2.06-20.53) | 0.002 |
| *Wood or bamboo walls* | |  |  |  |  |
|  | No | 1957 | 817 | Ref |  |
|  | Yes | 8143 | 4320 | 8.96 (2.29-35.03) | 0.002 |
| *Household farms palm oil* | |  |  |  |  |
|  | No | 9106 | 4509 | Ref |  |
|  | Yes | 994 | 628 | 18.66 (3.11-112.10) | 0.003 |
| *Insect screens in house* | |  |  |  |  |
|  | No | 9410 | 4697 | Ref |  |
|  | Yes | 690 | 440 | 22.73 (3.29-157.16) | 0.003 |
| *Individual engages in farm work* | |  |  |  |  |
|  | No | 8075 | 3823 | Ref |  |
|  | Yes | 2025 | 1314 | 2.56 (1.34-4.91) | 0.004 |
| *Usually bathe outside* | |  |  |  |  |
|  | No | 7510 | 2091 | Ref |  |
|  | Yes | 2590 | 892 | 3.81 (1.51-9.60) | 0.005 |
| *Ethnicity* |  |  |  |  |  |
|  | Bajau | 884 | 300 | Ref |  |
|  | Dusun | 5074 | 2569 | 12.53 (2.65-59.15) |  |
|  | Other | 1050 | 522 | 6.74 (1.16-39.12) |  |
|  | Rungus | 2682 | 1541 | 23.46 (4.45-123.61) |  |
|  | Sungoi | 410 | 205 | 4.96 (0.34-72.76) | 0.006 |
| *Household has pet monkey* | |  |  |  |  |
|  | No | 9863 | 4953 | Ref |  |
|  | Yes | 237 | 184 | 73.16 (4.78-1120.33) | 0.007 |
| *Household has cattle* | |  |  |  |  |
|  | No | 9907 | 4995 | Ref |  |
|  | Yes | 193 | 142 | 63.18 (3.38-1179.67) | 0.015 |
| *Treatment- seeking behaviour during fever: obtain medcines from clinic* | | | | |  |
|  | No | 7085 | 3405 | Ref |  |
|  | Yes | 3015 | 1732 | 3.02 (1.24-7.37) | 0.015 |
| *Usually bathe at river* | |  |  |  |  |
|  | No | 9565 | 2752 | Ref |  |
|  | Yes | 535 | 231 | 9.31 (1.52-57.18) |  |
|  | Yes | 89 | 73 | 129.96 (2.93-5767.08) | 0.021 |
| *Usually walk to work* | |  |  |  |  |
|  | No | 7490 | 3581 | Ref |  |
|  | Yes | 2610 | 1556 | 1.88 (1.05-3.40) | 0.035 |
| *Floors in house made of concrete or tile* | |  |  |  |  |
|  | No | 6854 | 3607 | Ref |  |
|  | Yes | 3246 | 1530 | 0.27 (0.08-0.94) | 0.041 |
| *Plastic containers of water seen near the house* | | |  |  |  |
|  | No | 7473 | 3713 | Ref |  |
|  | Yes | 2627 | 1424 | 3.97 (1.06-14.82) | 0.042 |
| *Treatment- seeking behaviour during fever: don't seek treatment* | | | |  |  |
|  | No | 8775 | 4623 | Ref |  |
|  | Yes | 1325 | 514 | 0.32 (0.10-1.00) | 0.050 |
| *Sleep under a bed net* | |  |  |  |  |
|  | No | 2170 | 958 | Ref |  |
|  | Yes | 7930 | 4179 | 2.44 (0.94-6.33) | 0.068 |
| *Household owns a buffalo* | |  |  |  |  |
|  | No | 9985 | 5048 | Ref |  |
|  | Yes | 115 | 89 | 50.45 (1.16-2184.72) | 0.073 |
| *Travel time to nearest hospital* | |  |  |  |  |
|  | Quartile 1 | 2467 | 1121 | Ref |  |
|  | Quartile 2 | 2599 | 1345 | 6.63 (1.33-33.05) |  |
|  | Quartile 3 | 2533 | 1344 | 3.74 (0.73-19.19) |  |
|  | Quartile 4 | 2501 | 1327 | 6.79 (1.35-34.19) | 0.077 |
| *Go to forest* | |  |  |  |  |
|  | No | 9345 | 4633 | Ref |  |
|  | Yes | 755 | 504 | 2.38 (0.88-6.45) | 0.085 |
| *Windows in the house can close* | |  |  |  |  |
|  | None | 1088 | 616 | Ref |  |
|  | Some | 4546 | 2422 | 0.55 (0.08-3.61) |  |
|  | All | 4466 | 2099 | 0.18 (0.03-1.18) | 0.094 |
| *Use smoke to prevent mosquitoes* | |  |  |  |  |
|  | No | 9242 | 4609 | Ref |  |
|  | Yes | 858 | 528 | 3.23 (0.80-13.14) | 0.099 |
| *Contact with monkeys* | |  |  |  |  |
|  | No | 5478 | 2589 | Ref |  |
|  | Yes | 4622 | 2548 | 1.74 (0.89-3.43) | 0.107 |
| *Contact with long-tailed macaques* | |  |  |  |  |
|  | No | 5478 | 2589 | Ref |  |
|  | Yes | 4622 | 2548 | 1.74 (0.89-3.43) | 0.107 |
| *Use insecticide* | |  |  |  |  |
|  | No | 5455 | 2676 | Ref |  |
|  | Yes | 4645 | 2461 | 1.94 (0.86-4.36) | 0.111 |
| *Go to forest at night (5pm-6am)* | |  |  |  |  |
|  | No | 9622 | 4803 | Ref |  |
|  | Yes | 478 | 334 | 2.67 (0.78-9.17) | 0.114 |
| *Go to forest at night (5pm-10pm)* | |  |  |  |  |
|  | No | 9664 | 4832 | Ref |  |
|  | Yes | 436 | 305 | 2.71 (0.74-9.86) | 0.125 |
| *Walk to work or school through forest* | |  |  |  |  |
|  | No | 9146 | 4544 | Ref |  |
|  | Yes | 954 | 593 | 1.96 (0.82-4.96) | 0.127 |
| *Age of house* | |  |  |  |  |
|  | 1 to 5 years | 1704 | 750 | Ref |  |
|  | Over 5 years | 7993 | 4184 | 4.74 (1.11-20.30) |  |
|  | Under 1 year | 368 | 178 | 0.72 (0.04-13.90) |  |
|  | Unknown | 35 | 25 | 31.12 (0.01-94274.66) | 0.130 |
| *Elevation (in metres above sea level)* | |  |  |  |  |
|  | Under 50 | 5701 | 2906 | Ref |  |
|  | 50-250 | 2248 | 1224 | 4.12 (0.98-17.29) |  |
|  | 250-500 | 1227 | 548 | 0.47 (0.08-2.91) |  |
|  | Over 500 | 924 | 459 | 1.56 (0.20-11.95) | 0.153 |
| *Household farms corn* | |  |  |  |  |
|  | No | 9854 | 4988 | Ref |  |
|  | Yes | 246 | 149 | 9.89 (0.41-240.08) | 0.182 |
| *Gaps in eaves of the house* | |  |  |  |  |
|  | No | 5561 | 2773 | Ref |  |
|  | Yes | 4539 | 2364 | 2.06 (0.64-6.60) | 0.223 |
| *Treatment- seeking behaviour during fever: go to hospital* | | | |  |  |
|  | No | 4820 | 2346 | Ref |  |
|  | Yes | 5280 | 2791 | 1.68 (0.72-3.88) | 0.228 |
| *Corrugated iron roof* | |  |  |  |  |
|  | No | 1136 | 561 | Ref |  |
|  | Yes | 8964 | 4576 | 3.00 (0.49-18.34) | 0.244 |
| *Kitchen outside house* | |  |  |  |  |
|  | No | 9661 | 4888 | Ref |  |
|  | Yes | 439 | 249 | 4.72 (0.34-65.62) | 0.260 |
| *Any early morning activities outside the house* | | |  |  |  |
|  | No | 9768 | 5016 | Ref |  |
|  | Yes | 332 | 121 | 0.40 (0.07-2.14) | 0.285 |
| *Travel to/or from work or school between 5pm and 10pm* | | | |  |  |
|  | No | 8448 | 4234 | Ref |  |
|  | Yes | 1652 | 903 | 1.47 (0.71-3.02) | 0.300 |
| *Hunting in forest* | |  |  |  |  |
|  | No | 9900 | 4993 | Ref |  |
|  | Yes | 200 | 144 | 2.42 (0.38 - 15.18) | 0.339 |
| *Use a fan to prevent mosquitoes* | |  |  |  |  |
|  | No | 8124 | 4051 | Ref |  |
|  | Yes | 1976 | 1086 | 1.64 (0.58 - 4.63) | 0.352 |
| *Length of time resident at current house* | | |  |  |  |
|  | 1 to 5 years | 1635 | 736 | Ref |  |
|  | Over 5 years | 8059 | 4190 | 3.38 (0.75 - 15.22) |  |
|  | Less than 1 year | 385 | 200 | 0.94 (0.05 - 18.43) |  |
|  | Unknown | 21 | 11 | 0.32 (0 - 3025.67) | 0.365 |
| *Monkeys seen around the village* | |  |  |  |  |
|  | No | 7433 | 3651 | Ref |  |
|  | Yes | 2667 | 1486 | 1.41 (0.67 - 2.97) | 0.370 |
| *Occupation* | |  |  |  |  |
|  | Farmer | 1412 | 902 | Ref |  |
|  | Fishing | 180 | 55 | 0.17 (0.02 - 1.34) |  |
|  | None | 4751 | 2273 | 0.46 (0.22 - 0.96) |  |
|  | Office/shop | 371 | 161 | 0.32 (0.07 - 1.39) |  |
|  | Other | 296 | 139 | 0.42 (0.09 - 2.06) |  |
|  | Palm oil plantation | 96 | 62 | 1.01 (0.07 - 14.56) |  |
|  | Rubber plantation | 254 | 164 | 1.28 (0.2 - 8.01) |  |
|  | Student | 2740 | 1381 | 0.53 (0.23 - 1.2) | 0.391 |
| *Don’t use any mosquito prevention* | |  |  |  |  |
|  | No | 9852 | 5015 | Ref |  |
|  | Yes | 248 | 122 | 0.38 (0.04 - 3.69) | 0.407 |
| *Collect wood in forest* | |  |  |  |  |
|  | No | 9870 | 4993 | Ref |  |
|  | Yes | 230 | 144 | 1.88 (0.35 - 10.02) | 0.456 |
| *Cleared land in the past year* | |  |  |  |  |
|  | No | 9870 | 4993 | Ref |  |
|  | Yes | 230 | 144 | 1.88 (0.35 - 10.02) | 0.456 |
| *Involved in construction in the past year* | | |  |  |  |
|  | No | 9870 | 4993 | Ref |  |
|  | Yes | 230 | 144 | 1.88 (0.35 - 10.02) | 0.456 |
| *Frequency of monkey sightings* | |  |  |  |  |
|  | Never | 5449 | 2570 | Ref |  |
|  | Daily | 1180 | 596 | 1.18 (0.35 - 3.96) |  |
|  | Monthly | 1774 | 995 | 1.85 (0.78 - 4.38) |  |
|  | Weekly | 1080 | 627 | 2.08 (0.73 - 5.95) |  |
|  | Yearly | 617 | 349 | 2.02 (0.56 - 7.33) | 0.457 |
| *Self-reported previous malaria diagnosis* | | |  |  |  |
|  | No | 8771 | 4385 | Ref |  |
|  | Yes | 1329 | 752 | 1.31 (0.63 - 2.73) | 0.468 |
| *Uncovered water containers seen around the house* | | |  |  |  |
|  | Unknown | 209 | 105 | Ref |  |
|  | No | 3518 | 1694 | 0.69 (0.02 - 31.38) |  |
|  | Yes | 6373 | 3338 | 1.47 (0.03 - 63.96) | 0.472 |
| *Travel to/from work or school between 11pm and 6am* | | | |  |  |
|  | No | 9211 | 4635 | Ref |  |
|  | Yes | 889 | 502 | 1.42 (0.52 - 3.91) | 0.490 |
| *Treatment- seeking behaviour during fever: take traditional medicines* | | | | |  |
|  | No | 9672 | 4893 | Ref |  |
|  | Yes | 428 | 244 | 1.76 (0.33 - 9.41) | 0.510 |
| *Report taking anti-malaria medication* | |  |  |  |  |
|  | No | 10026 | 5089 | Ref |  |
|  | Yes | 74 | 48 | 2.28 (0.13 - 40.93) | 0.575 |
| *Have stayed outside the village in the past month* | | |  |  |  |
|  | No | 9678 | 4893 | Ref |  |
|  | Yes | 422 | 244 | 1.47 (0.35 - 6.09) | 0.598 |
| *Go to forest at night (11pm-6am)* | |  |  |  |  |
|  | No | 10016 | 5078 | Ref |  |
|  | Yes | 84 | 59 | 2.03 (0.13 - 31.66) | 0.609 |
| *Well observed near house* | |  |  |  |  |
|  | No | 8435 | 4269 | Ref |  |
|  | Yes | 1665 | 868 | 1.47 (0.3 - 7.13) | 0.631 |
| *Occupation place* | |  |  |  |  |
|  | In district | 1864 | 1010 | Ref |  |
|  | Around the house | 5538 | 2671 | 0.71 (0.34 - 1.46) |  |
|  | In village | 2586 | 1406 | 0.99 (0.43 - 2.26) |  |
|  | Different district | 112 | 50 | 0.64 (0.05 - 7.38) | 0.642 |
| *Treatment- seeking behaviour during fever sees traditional healer* | | | |  |  |
|  | No | 10086 | 5127 | Ref |  |
|  | Yes | 14 | 10 | 4.59 (0.01 - 3300.9) | 0.649 |
| *House has a toilet* | |  |  |  |  |
|  | No | 1279 | 645 | Ref |  |
|  | Yes | 8821 | 4492 | 1.4 (0.26 - 7.49) | 0.698 |
| *Use window screens to prevent mosquitoes* | | |  |  |  |
|  | No | 10044 | 5103 | Ref |  |
|  | Yes | 56 | 34 | 1.63 (0.05 - 56.7) | 0.787 |
| *Lake near house* | |  |  |  |  |
|  | No | 10018 | 5099 | Ref |  |
|  | Yes | 82 | 38 | 0.47 (0 - 227.75) | 0.813 |
| *Have slept outside walls (out of houses) in the past month* | | | |  |  |
|  | No | 10050 | 5106 | Ref |  |
|  | Yes | 50 | 31 | 1.45 (0.05 - 46.5) | 0.831 |
| *Pond near house* | |  |  |  |  |
|  | No | 8157 | 4106 | Ref |  |
|  | Yes | 1943 | 1031 | 0.86 (0.2 - 3.72) | 0.845 |
| *Monkeys seen around the house* | |  |  |  |  |
|  | No | 8816 | 4478 | Ref |  |
|  | Yes | 1284 | 659 | 0.93 (0.3 - 2.89) | 0.904 |
| *Other activities in evenings* | |  |  |  |  |
|  | Fishing | 263 | 162 | Ref |  |
|  | None | 7332 | 3707 | 0.58 (0.11 - 3) |  |
|  | Other | 491 | 249 | 0.65 (0.08 - 4.98) |  |
|  | Visiting outside house | 1403 | 679 | 0.5 (0.08 - 2.93) |  |
|  | Sport | 611 | 340 | 0.81 (0.12 - 5.39) | 0.911 |
| *Gender* |  |  |  |  |  |
|  | No | 5324 | 2674 | Ref |  |
|  | Yes | 4776 | 2463 | 1.02 (0.65 - 1.6) | 0.938 |
| *Where are stays outside of the village* | |  |  |  |  |
|  | None | 9686 | 4896 | Ref |  |
|  | District | 241 | 148 | 1.65 (0.25 - 11.06) |  |
|  | Forest | 17 | 10 | 2.31 (0.01 - 827.87) |  |
|  | Outside | 145 | 76 | 1.06 (0.11 - 10.38) |  |
|  | Plantation | 11 | 7 | 7.05 (0 - 1150929.4) | 0.975 |
| *Age* |  |  |  |  |  |
|  | Under 5 | 1160 | 552 | Ref |  |
|  | 5-15 | 2611 | 1295 | 1.1 (0.47 - 2.61) |  |
|  | 15-30 | 1944 | 996 | 1.17 (0.48 - 2.85) |  |
|  | 30-55 | 2833 | 1472 | 1.16 (0.5 - 2.68) |  |
|  | Over 55 | 1552 | 822 | 1.11 (0.41 - 3.04) | 0.997 |

**Supplemental Table 3. Crude odds ratios for fixed effects for household and individual level factors impact the odds of poultry exposure.** Variables with p$\leq$0.2 were included in the development of a final model. This model was made with the lme4 package*(1)* and analysed with the broom.mixed package(2) in RStudio version 1.3.1093*(3, 4)*.

| ***Variable*** |  | **Total number** | **Number of swine owners** | **Crude odds ratios (95% CI)** | **P value** |
| --- | --- | --- | --- | --- | --- |
| *Household collects wood from forest* | | |  |  |  |
|  | No | 7716 | 126 | Ref |  |
|  | Yes | 2384 | 103 | 3.07 (0.24 - 39.09) | < 0.001 |
| *Piped water inside house* | |  |  |  |  |
|  | No | 4296 | 161 | Ref |  |
|  | Yes | 5804 | 68 | 0.34 (0.02 - 5.11) | < 0.001 |
| *Treatment- seeking behaviour during fever: don't seek treatment* | | | | |  |
|  | No | 8775 | 216 | Ref |  |
|  | Yes | 1325 | 13 | 0 (0 - 0) | < 0.001 |
| *Insect screens in house* | |  |  |  |  |
|  | No | 9410 | 211 | Ref |  |
|  | Yes | 690 | 18 | 0 (0 - 0) | < 0.001 |
| *Ethnicity* |  |  |  |  |  |
|  | Bajau | 884 | 7 | Ref |  |
|  | Dusun | 5074 | 91 | 1.96 (0 - 46019693.34) |  |
|  | Other | 1050 | 18 | 1.29 (0 - 2060545692) |  |
|  | Rungus | 2682 | 113 | 3.94 (0 - 389054860.6) |  |
|  | Sungoi | 410 | 0 | 0 (0 - Inf) | < 0.001 |
| *House height* | |  |  |  |  |
|  | Less than 1m | 2094 | 63 | Ref |  |
|  | Over 1m | 4416 | 121 | 1.96 (0.05 - 82.13) |  |
|  | Over water | 112 | 0 | 0 (0 - 3.02e+284) | 0.01 |
| *Age of house* | |  |  |  |  |
|  | 1 to 5 years | 1704 | 19 | Ref |  |
|  | Over 5 years | 7993 | 177 | 3.04 (0 - 4.72e+20) |  |
|  | Under 1 year | 368 | 33 | 9.87 (0 - 8.08e+24) |  |
|  | Unknown | 35 | 0 | 0 (0 - Inf) | 0.258 |
| *House has a toilet* | |  |  |  |  |
|  | No | 1279 | 63 | Ref |  |
|  | Yes | 8821 | 166 | 0.37 (0.02 - 8.11) | 0.563 |
| *Household farms rubber* | |  |  |  |  |
|  | No | 6564 | 87 | Ref |  |
|  | Yes | 3536 | 142 | 2.2 (0.14 - 34.3) | 0.577 |
| *Household collects food from the forest* | | |  |  |  |
|  | No | 8074 | 154 | Ref |  |
|  | Yes | 2026 | 75 | 2.29 (0.13 - 39.92) | 0.588 |
| *Household owns a goat* | |  |  |  |  |
|  | No | 10011 | 217 | Ref |  |
|  | Yes | 89 | 12 | 7.75 (0.02 - 2991.66) | 0.604 |
| *Monkeys raid household crops* | | |  |  |  |
|  | No | 9006 | 172 | Ref |  |
|  | Yes | 1094 | 57 | 2.56 (0.1 - 66.61) | 0.606 |
| *Toilet is inside house* | |  |  |  |  |
|  | No | 5787 | 174 | Ref |  |
|  | Yes | 4313 | 55 | 0.47 (0.02 - 10.56) | 0.616 |
| *Go to forest at night (5pm-10pm)* | | |  |  |  |
|  | No | 9664 | 201 | Ref |  |
|  | Yes | 436 | 28 | 2.92 (0.07 - 126.62) | 0.616 |
| *Individual engages in farmwork* | | |  |  |  |
|  | No | 8075 | 151 | Ref |  |
|  | Yes | 2025 | 78 | 1.95 (0.15 - 26.04) | 0.627 |
| *Floors in house made of concrete or tile* | | | |  |  |
|  | No | 6854 | 189 | Ref |  |
|  | Yes | 3246 | 40 | 0.44 (0.01 - 16.25) | 0.629 |
| *Go to forest at night (5pm-6am)* | | |  |  |  |
|  | No | 9622 | 201 | Ref |  |
|  | Yes | 478 | 28 | 2.71 (0.06 - 114.73) | 0.637 |
| *Go to forest* | |  |  |  |  |
|  | No | 9345 | 191 | Ref |  |
|  | Yes | 755 | 38 | 2.34 (0.08 - 66.38) | 0.646 |
| *Household collects medicine from the forest* | | | |  |  |
|  | No | 8627 | 161 | Ref |  |
|  | Yes | 1473 | 68 | 2.12 (0.09 - 48.55) | 0.66 |
| *Usually bathe outside* | |  |  |  |  |
|  | No | 7510 | 2091 | Ref |  |
|  | Yes | 2590 | 892 | 1.88 (0.12 - 29.37) | 0.661 |
| *Sea near the house* | |  |  |  |  |
|  | No | 9262 | 227 | Ref |  |
|  | Yes | 838 | 2 | 0.16 (0 - 13976.36) | 0.663 |
| *Treatment- seeking behaviour during fever: take traditional medicines* | | | | |  |
|  | No | 9672 | 213 | Ref |  |
|  | Yes | 428 | 16 | 2.5 (0.03 - 231.56) | 0.725 |
| *Socioeconomic status* | |  |  |  |  |
|  | Quartile 1 | 2251 | 109 | Ref |  |
|  | Quartile 2 | 2526 | 70 | 0.45 (0.02 - 10.97) |  |
|  | Quartile 3 | 2626 | 31 | 0.18 (0 - 14.5) |  |
|  | Quartile 4 | 2697 | 19 | 0.11 (0 - 23.05) | 0.728 |
| *Household has cattle* | |  |  |  |  |
|  | No | 9907 | 219 | Ref |  |
|  | Yes | 193 | 10 | 3.23 (0.01 - 1205.04) | 0.741 |
| *Treatment- seeking behaviour during fever: obtain medcines from clinic* | | | | |  |
|  | No | 7085 | 131 | Ref |  |
|  | Yes | 3015 | 98 | 1.6 (0.11 - 24.16) | 0.741 |
| *Self-reported previous malaria diagnosis* | | | |  |  |
|  | No | 8771 | 189 | Ref |  |
|  | Yes | 1329 | 40 | 1.71 (0.08 - 37.01) | 0.745 |
| *Usually walk to work* | |  |  |  |  |
|  | No | 7490 | 150 | Ref |  |
|  | Yes | 2610 | 79 | 1.53 (0.12 - 19.72) | 0.748 |
| *Walk to work or school through forest* | | |  |  |  |
|  | No | 9146 | 191 | Ref |  |
|  | Yes | 954 | 38 | 1.77 (0.06 - 49.84) | 0.75 |
| *Hunting in forest* | |  |  |  |  |
|  | No | 9900 | 217 | Ref |  |
|  | Yes | 200 | 12 | 2.65 (0.01 - 674.02) | 0.755 |
| *Report taking anti-malaria medication* | | |  |  |  |
|  | No | 10026 | 222 | Ref |  |
|  | Yes | 74 | 7 | 3.68 (0 - 11132.23) | 0.784 |
| *Gaps in eaves of the house* | | |  |  |  |
|  | No | 5561 | 113 | Ref |  |
|  | Yes | 4539 | 116 | 1.42 (0.09 - 22.31) | 0.803 |
| *Swidden farming* | |  |  |  |  |
|  | No | 5338 | 84 | Ref |  |
|  | Yes | 4762 | 145 | 1.39 (0.09 - 22.07) | 0.813 |
| *Travel to/or from work or school between 5pm and 10pm* | | | | |  |
|  | No | 8448 | 185 | Ref |  |
|  | Yes | 1652 | 44 | 1.39 (0.07 - 29.69) | 0.837 |
| *Have stayed outside the village in the past month* | | | |  |  |
|  | No | 9678 | 225 | Ref |  |
|  | Yes | 422 | 4 | 0.47 (0 - 2165.03) | 0.849 |
| *Travel to/from work or school between 11pm and 6am* | | | | |  |
|  | No | 9211 | 214 | Ref |  |
|  | Yes | 889 | 15 | 0.62 (0 - 111.95) | 0.851 |
| *Lake near house* | |  |  |  |  |
|  | No | 10018 | 229 | Ref |  |
|  | Yes | 82 | 0 | 0 (0 - 5.06e+299) | 0.854 |
| *Contact with monkeys* | |  |  |  |  |
|  | No | 5478 | 116 | Ref |  |
|  | Yes | 4622 | 113 | 1.26 (0.1 - 16.21) | 0.858 |
| *Contact with long-tailed macaques* | | |  |  |  |
|  | No | 5478 | 116 | Ref |  |
|  | Yes | 4622 | 113 | 1.26 (0.1 - 16.21) | 0.858 |
| *Go to forest at night (11pm-6am)* | | |  |  |  |
|  | No | 10016 | 227 | Ref |  |
|  | Yes | 84 | 2 | 2.47 (0 - 28089.58) | 0.868 |
| *Household farms palm oil* | | |  |  |  |
|  | No | 9106 | 186 | Ref |  |
|  | Yes | 994 | 43 | 1.41 (0.02 - 83.47) | 0.875 |
| *Monkeys seen around the house* | | |  |  |  |
|  | No | 8816 | 205 | Ref |  |
|  | Yes | 1284 | 24 | 0.72 (0.01 - 60.39) | 0.881 |
| *Usually bathe at river* | |  |  |  |  |
|  | No | 9565 | 2752 | Ref |  |
|  | Yes | 535 | 231 | 1.43 (0.01 - 230.68) | 0.896 |
| *Wood or bamboo walls* | |  |  |  |  |
|  | No | 1957 | 37 | Ref |  |
|  | Yes | 8143 | 192 | 1.27 (0.03 - 53.91) | 0.898 |
| *Treatment- seeking behaviour during fever: go to hospital* | | | | |  |
|  | No | 4820 | 106 | Ref |  |
|  | Yes | 5280 | 123 | 1.16 (0.08 - 17.22) | 0.914 |
| *Don’t use any mosquito prevention* | | |  |  |  |
|  | No | 9852 | 228 | Ref |  |
|  | Yes | 248 | 1 | 0.57 (0 - 55828.34) | 0.917 |
| *Household farms corn* | |  |  |  |  |
|  | No | 9854 | 227 | Ref |  |
|  | Yes | 246 | 2 | 0.58 (0 - 56127.47) | 0.919 |
| *Monkeys seen around the village* | | |  |  |  |
|  | No | 7433 | 166 | Ref |  |
|  | Yes | 2667 | 63 | 1.16 (0.07 - 19.64) | 0.92 |
| *Windows in the house can close* | | |  |  |  |
|  | None | 1088 | 37 | Ref |  |
|  | Some | 4546 | 98 | 0.69 (0.02 - 31.5) |  |
|  | All | 4466 | 94 | 0.44 (0.01 - 26.6) | 0.922 |
| *Well observed near house* | |  |  |  |  |
|  | No | 8435 | 178 | Ref |  |
|  | Yes | 1665 | 51 | 1.19 (0.04 - 39.91) | 0.925 |
| *Kitchen outside house* | |  |  |  |  |
|  | No | 9661 | 222 | Ref |  |
|  | Yes | 439 | 7 | 1.34 (0 - 487.22) | 0.926 |
| *Household has pet monkey* | | |  |  |  |
|  | No | 9863 | 226 | Ref |  |
|  | Yes | 237 | 3 | 0.61 (0 - 58405.55) | 0.926 |
| *Use a fan to prevent mosquitoes* | | |  |  |  |
|  | No | 8124 | 173 | Ref |  |
|  | Yes | 1976 | 56 | 1.16 (0.04 - 30.49) | 0.93 |
| *Household has livestock* | |  |  |  |  |
|  | No | 10082 | 229 | Ref |  |
|  | Yes | 18 | 0 | 0 (0 - 4.53e+213) | 0.932 |
| *Distance of farming land from the house* | | | |  |  |
|  | Near the house | 2110 | 108 | Ref |  |
|  | Same village | 3810 | 76 | 0.44 (0.02 - 11.54) |  |
|  | No farmland | 3747 | 34 | 0.32 (0.01 - 11.63) |  |
|  | Outside the village | 433 | 11 | 0.7 (0 - 316.52) | 0.933 |
| *Use smoke to prevent mosquitoes* | | |  |  |  |
|  | No | 9242 | 206 | Ref |  |
|  | Yes | 858 | 23 | 1.22 (0.01 - 116.02) | 0.934 |
| *Treatment- seeking behaviour during fever sees traditional healer* | | | | |  |
|  | No | 10086 | 229 | Ref |  |
|  | Yes | 14 | 0 | 0 (0 - Inf) | 0.94 |
| *Household farms vegetables* | | |  |  |  |
|  | No | 8454 | 198 | Ref |  |
|  | Yes | 1646 | 31 | 0.87 (0.02 - 42.9) | 0.942 |
| *Travel time to nearest hospital* | | |  |  |  |
|  | Quartile 1 | 2467 | 45 | Ref |  |
|  | Quartile 2 | 2599 | 84 | 2.34 (0.04 - 136.22) | 0.944 |
|  | Quartile 3 | 2533 | 30 | 0.79 (0 - 128.86) | 0.944 |
|  | Quartile 4 | 2501 | 70 | 1.98 (0.03 - 130.83) | 0.944 |
| *Pond near house* | |  |  |  |  |
|  | No | 8157 | 186 | Ref |  |
|  | Yes | 1943 | 43 | 0.88 (0.02 - 33.18) | 0.946 |
| *Household has rice paddies* | | |  |  |  |
|  | No | 8791 | 215 | Ref |  |
|  | Yes | 1309 | 14 | 0.87 (0.01 - 63.73) | 0.947 |
| *Length of time resident at current house* | | | |  |  |
|  | 1 to 5 years | 1635 | 32 | Ref |  |
|  | Over 5 years | 8059 | 164 | 1.53 (0.02 - 145.93) |  |
|  | Less than 1 year | 385 | 33 | 5.77 (0.02 - 1799.5) |  |
|  | Unknown | 21 | 0 | 0 (0 - 1.07e+209) | 0.948 |
| *Distance to nearest clinic* | |  |  |  |  |
|  | Quartile 1 | 2495 | 26 | Ref |  |
|  | Quartile 2 | 2592 | 30 | 0.88 (0.01 - 124.97) |  |
|  | Quartile 3 | 2420 | 83 | 2.1 (0.03 - 140.13) |  |
|  | Quartile 4 | 2593 | 90 | 2.32 (0.04 - 138.36) | 0.95 |
| *Gender* |  |  |  |  |  |
|  | Male | 5324 | 128 | Ref |  |
|  | Female | 4776 | 101 | 0.94 (0.09 - 10.19) | 0.96 |
| *Corrugated iron roof* | |  |  |  |  |
|  | No | 1136 | 28 | Ref |  |
|  | Yes | 8964 | 201 | 1.12 (0.01 - 104.84) | 0.961 |
| *Household farms fruit* | |  |  |  |  |
|  | No | 8527 | 194 | Ref |  |
|  | Yes | 1573 | 35 | 0.92 (0.02 - 45.47) | 0.966 |
| *River near the house* | |  |  |  |  |
|  | No | 4911 | 132 | Ref |  |
|  | Yes | 5189 | 97 | 0.97 (0.06 - 15.1) | 0.98 |
| *Sleep under a bed net* | |  |  |  |  |
|  | No | 2170 | 57 | Ref |  |
|  | Yes | 7930 | 172 | 0.96 (0.04 - 23.98) | 0.98 |
| *Where are stays outside of the village* | | |  |  |  |
|  | No | 10050 | 228 | Ref |  |
|  | Yes | 50 | 1 | 0.8 (0 - 85397043.61) | 0.981 |
| *Household has pet monkey* | | |  |  |  |
|  | No | 8267 | 196 | Ref |  |
|  | Yes | 1833 | 33 | 1.04 (0.03 - 35.02) | 0.983 |
| *Elevation (in metres above sea level)* | | |  |  |  |
|  | Under 50 | 5701 | 128 | Ref |  |
|  | 50-250 | 2248 | 65 | 1.55 (0.07 - 34.13) |  |
|  | 250-500 | 1227 | 24 | 1.03 (0.01 - 88.7) |  |
|  | Over 500 | 924 | 12 | 0.51 (0 - 472.71) | 0.986 |
| *Use insecticide* | |  |  |  |  |
|  | No | 5455 | 125 | Ref |  |
|  | Yes | 4645 | 104 | 0.98 (0.07 - 14.19) | 0.989 |
| *Frequency of monkey sightings* | |  |  |  |  |
|  | Never | 5449 | 116 | Ref |  |
|  | Daily | 1180 | 11 | 0.62 (0 - 142.15) |  |
|  | Monthly | 1774 | 66 | 1.82 (0.09 - 37.39) |  |
|  | Weekly | 1080 | 23 | 1.24 (0.02 - 74.15) |  |
|  | Yearly | 617 | 13 | 0.81 (0 - 279.79) | 0.994 |
| *Age* |  |  |  |  |  |
|  | Under 5 | 1160 | 20 | Ref |  |
|  | 5-15 | 2611 | 55 | 1.13 (0.01 - 153.27) |  |
|  | 15-30 | 1944 | 43 | 1.44 (0.01 - 215.61) |  |
|  | 30-55 | 2833 | 63 | 1.56 (0.01 - 173.95) |  |
|  | Over 55 | 1552 | 48 | 2.44 (0.02 - 331.93) | 0.995 |
| *Occupation place* | |  |  |  |  |
|  | In district | 1864 | 41 | Ref |  |
|  | Around the house | 5538 | 122 | 1.22 (0.03 - 44.09) |  |
|  | In village | 2586 | 64 | 1.46 (0.03 - 73.62) |  |
|  | Different district | 112 | 2 | 0.88 (0 - 1035585.04) | 0.998 |
| *Plastic containers of water seen near the house* | | | |  |  |
|  | No | 7473 | 181 | Ref |  |
|  | Yes | 2627 | 48 | 1 (0.04 - 22.83) | 0.998 |
| *Occupation* |  |  |  |  |  |
|  | Farmer | 1412 | 58 | Ref |  |
|  | Fishing | 180 | 1 | 0.23 (0 - 250893.79) |  |
|  | None | 4751 | 95 | 0.47 (0.03 - 8.65) |  |
|  | Office/shop | 371 | 6 | 0.30 (0 - 1280.59) |  |
|  | Other | 296 | 6 | 0.49 (0 - 994.26) |  |
|  | Palm oil plantation | 96 | 3 | 0.43 (0 - 128725.39) |  |
|  | Rubber plantation | 254 | 5 | 0.34 (0 - 3219.28) |  |
|  | Student | 2740 | 55 | 0.37 (0.01 - 12.6) | 1.00 |
| *Other activities in evenings* | | |  |  |  |
|  | Fishing | 263 | 8 | Ref |  |
|  | None | 7332 | 163 | 0.7 (0 - 668.06) |  |
|  | Other | 491 | 10 | 0.81 (0 - 4462.41) |  |
|  | Visiting outside house | 1403 | 34 | 0.97 (0 - 1592.45) |  |
|  | Sport | 611 | 14 | 0.7 (0 - 3263.52) | 1 |
| *Where are stays outside of the village* | | |  |  |  |
|  | None | 9686 | 223 | Ref |  |
|  | District | 241 | 2 | 0.41 (0 - 1112957085) |  |
|  | Forest | 17 | 1 | 2.14 (0 - 1.23e+17) |  |
|  | Outside | 145 | 3 | 0.81 (0 - 156894861.4) |  |
|  | Plantation | 11 | 0 | 0 (0 - Inf) | 1 |
| *Collect wood in forest* | |  |  |  |  |
|  | No | 9870 | 221 | Ref |  |
|  | Yes | 230 | 8 | 0 (0 - 0) | 1 |
| *Cleared land in the past year* | | |  |  |  |
|  | No | 9870 | 221 | Ref |  |
|  | Yes | 230 | 8 | 0 (0 - 0) | 1 |
| *Involved in construction in the past year* | | | |  |  |
|  | No | 9870 | 221 | Ref |  |
|  | Yes | 230 | 8 | 0 (0 - 0) | 1 |
| *Household owns a buffalo* | | |  |  |  |
|  | No | 9985 | 216 | Ref |  |
|  | Yes | 115 | 13 | 0 (0 - 0) | 1 |

**Supplemental Table 4. Crude odds ratios for fixed effects for household and individual level factors impact the odds of poultry exposure.** Variables with p$\leq$0.2 were included in the development of a final model. This model was made with the lme4 package*(1)* and analysed with the broom.mixed package(2) in RStudio version 1.3.1093*(3, 4)*.

# Multivariate risk factor analysis

| **Odds of NHP sightings** | | | | | | |  | | | |
| --- | --- | --- | --- | --- | --- | --- | --- | --- | --- | --- |
| Household-level risk factors | | | | | | | Individual-level risk factors | | | |
| Predictor variable | Adjusted Odds Ratios | | 95% CI | | | P value | Predictor variable | Adjusted Odds Ratios | 95% CI | P value |
| *House near sea* | | | | | | | *Age* | | | |
| No | Ref | |  | | |  | Under 5 | Ref |  |  |
| Yes | 11.70 | | 7.70 – 17.80 | | | <0.001 | 5-15 | 1.83 | 1.21 – 2.75 |  |
| *Travel time to nearest hospital* | | | | | | | 15-30 | 2.38 | 1.56 – 3.65 |  |
| Quartile 1 | Ref | |  | | |  | 30-55 | 2.85 | 1.89 – 4.30 |  |
| Quartile 2 | 2.14 | | 1.34 – 3.39 | | |  | Over 55 | 2.95 | 1.86 – 4.67 | <0.001 |
| Quartile 3 | 1.71 | | 1.06 – 2.77 | | |  | *Treatment-seeking behavior during fever: go to hospital* | | | |
| Quartile 4 | 3.92 | | 2.45 – 6.26 | | | <0.001 | No | Ref |  |  |
| *Household elevation (in meters above sea level)* | | | | | | | Yes | 0.56 | 0.42 – 0.74 | <0.001 |
| Under 50 | | Ref |  | |  | | *Engages in farm work* | | | |
| 50-250 | | 0.30 | 0.20 – 0.45 | |  | | No | Ref |  |  |
| 250-500 | | 0.23 | 0.13 – 0.42 | |  | | Yes | 1.45 | 1.09 – 1.92 | **0.011** |
| Over 500 | 0.18 | | 0.09 – 0.39 | | | <0.001 |  | | | |
| *Household farms corn* | | | | | | |  |  |  |  |
| No | Ref | |  | | |  |  |  |  |  |
| Yes | | 4.04 | 1.83 – 8.92 | 0.001 | | |  |  |  |  |
| *Gaps in eaves of house* | | | | | | |  |  |  |  |
| No | Ref | |  | | |  |  |  |  |  |
| Yes | 2.50 | | 1.79 – 3.50 | | | **<0.001** |  |  |  |  |
| *Number of windows in house that can close* | | | | | | |  |  |  |  |
| None | Ref | |  | | |  |  |  |  |  |
| Some | 2.43 | | 1.42 – 4.14 | | |  |  |  |  |  |
| All | 2.28 | | 1.32 – 3.93 | | | 0.003 |  |  |  |  |
| *Household collects wood from forest* | | | | | | |  |  |  |  |
| No | Ref | |  | | |  |  |  |  |  |
| Yes | 2.02 | | 1.11 – 3.65 | | | **0.021** |  |  |  |  |
| *Household farms fruit* | | | | | | |  |  |  |  |
| No | Ref | |  | | |  |  |  |  |  |
| Yes | 1.75 | | 1.14 – 2.70 | | | **0.011** |  |  |  |  |

**Supplemental Table 5. Adjusted odds ratios for fixed effects for household and individual level factors impact the odds of sighting an NHP.** The combination of predictors which yielded the model with the highest log likelihood is shown (-2157.854) with household as the random effect. A univariate analysis was first used to select from 95 possible explanatory variables; those with p$\leq$0.2 were included in the development of a final model. This model was made with the lme4 package*(1)* and analysed with the broom.mixed package(2) in RStudio version 1.3.1093*(3, 4)*.

| **Odds of bat sightings** | | | |  |  |  |  |
| --- | --- | --- | --- | --- | --- | --- | --- |
| Household-level risk factors | | | | Individual-level risk factors | | | |
| Predictor variable | Adjusted Odds Ratios | 95% CI | P value | Predictor variable | Adjusted Odds Ratios | 95% CI | P value |
| *House near river* | | | | *Age* |  |  |  |
| No | Ref |  |  | Under 5 | Ref |  |  |
| Yes | 6.59 | 4.52 – 9.62 | <0.001 | 5-15 | 3.84 | 2.73 – 5.41 |  |
| *Travel time to nearest clinic* | | | | 15-30 | 4.93 | 3.44 – 7.06 |  |
| Quartile 1 | Ref |  |  | 30-55 | 6.59 | 4.61 – 9.40 |  |
| Quartile 2 | 1.69 | 1.03 – 2.79 |  | Over 55 | 7.11 | 4.71 – 10.74 | <0.001 |
| Quartile 3 | 0.64 | 0.38 – 1.06 |  | *Frequency of monkey sightings* | | | |
| Quartile 4 | 1.17 | 0.71 – 1.92 | 0.002 | Never | Ref |  |  |
| *Household elevation (in meters above sea level)* | | | | Daily | 4.14 | 2.73 – 6.28 |  |
| Under 50 | Ref |  |  | Weekly | 6.44 | 4.69 – 8.83 |  |
| 50-250 | 4.75 | 2.99 – 7.54 |  | Monthly | 10.93 | 7.45 – 16.02 |  |
| 250-500 | 25.93 | 14.28 – 47.08 |  | Yearly | 13.09 | 8.03 – 21.33 | <0.001 |
| Over 500 | 4.97 | 2.61 – 9.49 | <0.001 | *Visits forest* | | | |
| *Distance of farmland from house* | | | | No | Ref |  |  |
| No farmland | Ref |  |  | Yes | 2.19 | 1.52 – 3.15 | <0.001 |
| Near the house | 1.76 | 1.07 – 2.90 |  | *Engages in farm work* | | | |
| Same village | 2.78 | 1.80 – 4.31 |  | No | Ref |  |  |
| Outside the village | 2.53 | 1.05 – 6.12 | <0.001 | Yes | 1.69 | 1.31 – 2.19 | <0.001 |
| *Water-filled plastic containers near house* | | | | *Activities in evenings* | | | |
| No | Ref |  |  | None | Ref |  |  |
| Yes | 5.71 | 3.78 – 8.62 | <0.001 | Visiting outside house | 0.73 | 0.54 – 1.00 |  |
| *House height* | | | | Sports | 1.97 | 1.37 – 2.85 |  |
| Less than 1m | Ref |  |  | Fishing | 1.97 | 1.12 – 3.47 |  |
| Over 1m | 2.8 | 1.90 – 4.14 |  | Other | 2.77 | 1.78 – 4.31 | <0.001 |
| Over water | 0.96 | 0.16 – 5.77 | <0.001 |  |  |  |  |
| *Monkeys seen around the house* | | | |  |  |  |  |
| No | Ref |  |  |  |  |  |  |
| Yes | 0.52 | 0.34 – 0.80 | 0.003 |  |  |  |  |
| *Wood or bamboo walls* | | | |  |  |  |  |
| No | Ref |  |  |  |  |  |  |
| Yes | 0.65 | 0.40 – 1.05 | 0.077 |  |  |  |  |

**Supplemental Table 6. Adjusted odds ratios for fixed effects for household and individual level factors impact the odds of sighting a bat**. Results are based on the binomial GLMM which yielded the highest log likelihood is shown (-4094.4) with household as the random effect. A univariate analysis was first used to select from 95 possible explanatory variables; those with p$\leq$0.2 were included in the development of a final model. This model was made with the lme4 package(1) and analysed with the broom.mixed package(2) in RStudio version 1.3.1093(3, 4).

| **Odds of poultry ownership** | | | |
| --- | --- | --- | --- |
| Household level risk factors | | | |
| Predictor variable | Adjusted Odds Ratios | 95% CI | P value |
| *Household farms rubber* | | | |
| No | Ref |  |  |
| Yes | 4.33 | 1.06– 17.61 | <0.001 |
| *Distance of farmland from house* | | | |
| No farmland | Ref |  |  |
| Near the house | 58.88 | 6.68 – 505.54 |  |
| Same village | 62.66 | 8.01 – 490.03 |  |
| Outside the village | 12.95 | 0.48 – 350.48 | <0.001 |
| *Household engages in swidden farming (slash-and-burn)* | | | |
| No | Ref |  | <0.001 |
| Yes | 41.11 | 7.90 – 213.78 |  |

**Supplemental Table 7. Adjusted odds ratios for fixed effects for household and individual level factors impact the odds of poultry ownership**. This binomial GLMM had the highest log likelihood (-2037.7) with household as the random effect. A univariate analysis was first used to select from 95 possible explanatory variables; those with p$\leq$0.2 were included in the development of a final model. This model was made with the lme4 package(1) and analysed with the broom.mixed package(2) in RStudio version 1.3.1093(3, 4).

| **Odds of swine ownership** | | | |
| --- | --- | --- | --- |
| Household level risk factors | | | |
| Predictor variable | Adjusted Odds Ratios | 95% CI | P value |
| *Piped water inside house* | | | |
| No | Ref |  |  |
| Yes | 0.36 | 0.02-5.11 | 0.433 |

**Supplemental Table 8. Adjusted odds ratios for fixed effects for household and individual level factors impact the odds of owning a swine.** This predictor yielded the best balance of high log likelihood (-272.94) and small confidence intervals in a binomial GLMM with household as the random effect. A univariate analysis was first used to select from 95 possible explanatory variables; those with p$\leq$0.2 were included in the development of a final model. This model was made with the lme4 package(1) and analysed with the broom.mixed package(2) in RStudio version 1.3.1093(3, 4).

# Univariate environmental risk factor analysis

|  |  |  |  |  |
| --- | --- | --- | --- | --- |
|  | **Bats** | **Swine** | **Poultry** | **NHPs** |
| Precipitation seasonality, 1970 - 2000 (coefficient of variation) | | | | |
|  | 2.20E-16 | 8.07E-14 | 1.14E-07 | 2.20E-16 |
| Distance from mangroves (m) | | | | |
|  | 2.20E-16 | 1.00E-11 | 1.81E-07 | 2.20E-16 |
| Distance from agricultural land (m) | | | | |
|  | 2.20E-16 | 1.75E-11 | 4.52E-07 | 2.20E-16 |
| Distance from the sea (m) | | | | |
|  | 2.20E-16 | 3.11E-09 | 2.23E-05 | 2.20E-16 |
| Mean diurnal range, 1970 - 2000 (°C) | | | | |
|  | 2.20E-16 | 1.88E-07 | 0.000648 | 2.20E-16 |
| Distance from old forest (primary) (m) | | | | |
|  | 2.20E-16 | 0.5894 | 0.0425 | 2.20E-16 |
| Minimum temperature of coldest month, 1970 - 2000 (°C) | | | | |
|  | 2.20E-16 | 0.000184 | 0.07408 | 2.20E-16 |
| Average temperature, 1970 - 2000 (°C) | | | | |
|  | 2.20E-16 | 0.003962 | 0.2812 | 2.20E-16 |
| Elevation (meters above sea level) | | | | |
|  | 4.90E-16 | 6.38E-03 | 0.0425 | 2.20E-16 |
| Euclidean distance from roads | | | | |
|  | 3.13E-05 | 0.005158 | 0.001676 | 1.77E-07 |
| Normalized differential vegetation index | | | | |
|  | 3.53E-05 | 2.20E-16 | 2.20E-16 | 2.20E-16 |
| Distance from oil palm plantation (m) | | | | |
|  | 8.55E-04 | 2.20E-16 | 0.000838 | 0.6793 |
| Aspect (degrees) | | | | |
|  | 4.95E-03 | 2.15E-05 | 0.7431 | 4.65E-02 |
| Distance from bush forest (secondary) (m) | | | | |
|  | 8.12E-03 | 0.02737 | 0.0011 | 2.20E-16 |
| Population density (per km^2^) | | | | |
|  | 0.02324 | 0.007705 | 0.7116 | 0.5195 |
| Precipitation of the wettest month. 1970 - 2000 (mm) | | | | |
|  | 9.72E-02 | 9.98E-16 | 5.17E-09 | 2.20E-16 |
| Distance from irrigated farmland (m) | | | | |
|  | 1.08E-01 | 0.07105 | 0.1813 | 2.20E-16 |
| Slope (degrees) | | | | |
|  | 0.6225 | 0.2372 | 0.8479 | 2.20E-16 |
| Maximum temperature of warmest month, 1970 - 2000 (°C) | | | | |
|  | 8.11E-01 | 0.5017 | 0.6617 | 2.20E-16 |
| Distance from rubber plantation (m) | | | | |
|  | 9.60E-01 | 0.3251 | 0.01879 | 2.20E-16 |

**Supplemental Table 9. P values from univariate analysis of environmental variables by species.** Binomial GLMMs were fit to understand factors which were associated with animal exposure. This model considered household as a random effect, as well as a variety of fixed effects adapted from questionnaire responses. A univariate analysis was first used to select from possible explanatory variables; those with p≤0.2 were included in the development of a final model. Log likelihood ratio tests were then used to identify the final model, through a parsimonious forward stepwise approach with a logit link fit.

# Multivariate environmental risk factor analysis

| **Odds of NHP sightings** | | | |
| --- | --- | --- | --- |
| Household-level risk factors | | | |
| Predictor variable | Adjusted Odds Ratios | 95% CI | P value |
| Normalized differential vegetation index | 1.16 | 1.14 – 1.19 | <0.001 |
| Population density (per km^2^) | 1.02 | 1.00 – 1.04 | 0.030 |
| Elevation (meters above sea level) | 0.43 | 0.31 – 0.59 | <0.001 |
| Aspect (degrees) | 0.98 | 0.97 – 1.00 | 0.007 |
| Distance from sea (m) | 0.03 | 0.03 – 0.04 | <0.001 |
| Average temperature, 1970 - 2000 (°C) | 0.00 | 0.00 – 0.00 | <0.001 |
| Median diurnal range, 1970 - 2000 (°C) | 0.17 | 0.12 – 0.24 | <0.001 |
| Maximum temperature of warmest month, 1970 - 2000 (°C) | 885.24 | 275.78 – 2847.14 | <0.001 |
| Minimum temperature of coldest month, 1970 - 2000 (°C) | 18.63 | 3.65 – 95.27 | <0.001 |
| Precipitation of the wettest month, 1970 - 2000 (mm) | 1.03 | 0.99 – 1.07 | 0.117 |
| Precipitation seasonality, 1970 - 2000 (coefficient of variation) | 0.28 | 0.24 – 0.32 | <0.001 |
| Distance from bush forest (secondary) (m) | 0.89 | 0.85 – 0.93 | <0.001 |
| Distance from mangroves (m) | 0.11 | 0.07 – 0.18 | <0.001 |
| Distance from rubber plantation (m) | 0.80 | 0.71 – 0.90 | <0.001 |
| Distance from agricultural land (m) | 49.72 | 28.91 – 85.56 | <0.001 |
| Distance from oil palm plantation (m) | 1.12 | 1.07 – 1.18 | <0.001 |
| Distance from irrigated farmland (m) | 1.09 | 1.02 – 1.17 | 0.008 |
| Distance from old forest (primary) (m) | 1.11 | 1.09 – 1.14 | <0.001 |

**Supplemental Table 10. Adjusted odds ratios for fixed effects for environmental and land-use factors impact the odds of sighting an NHP.** The combination of predictors which yielded the model with the highest log likelihood is shown (-20172.83) with household as the random effect. A univariate analysis was first used to select from possible explanatory variables; those with p$\leq$0.2 were included in the development of a final model. This model was made with the lme4 package(1) and analysed with the broom.mixed package(2) in RStudio version 1.3.1093(3, 4).

| **Odds of bat sightings** | | | |
| --- | --- | --- | --- |
| Household-level risk factors | | | |
| Predictor variable | Adjusted Odds Ratios | 95% CI | P value |
| Population density (per km^2^) | 0.91 | 0.88 – 0.94 | <0.001 |
| Elevation (meters above sea level) | 0.60 | 0.44 – 0.82 | 0.002 |
| Slope (degrees) | 0.89 | 0.86 – 0.92 | <0.001 |
| Aspect (degrees) | 1.06 | 1.04 – 1.07 | <0.001 |
| Distance from sea (m) | 2.33 | 1.85 – 2.92 | <0.001 |
| Average temperature, 1970 - 2000 (°C) | 0.01 | 0.00 – 0.09 | <0.001 |
| Median diurnal range, 1970 - 2000 (°C) | 0.43 | 0.30 – 0.62 | <0.001 |
| Maximum temperature of warmest month, 1970 - 2000 (°C) | 19.68 | 5.89 – 65.80 | <0.001 |
| Minimum temperature of coldest month, 1970 - 2000 (°C) | 12.23 | 2.18 – 68.72 | 0.004 |
| Precipitation of the wettest month, 1970 - 2000 (mm) | 1.15 | 1.11 – 1.20 | <0.001 |
| Precipitation seasonality, 1970 - 2000 (coefficient of variation) | 0.60 | 0.52 – 0.70 | <0.001 |
| Distance from bush forest (secondary) (m) | 1.14 | 1.10 – 1.19 | <0.001 |
| Distance from mangroves (m) | 21.80 | 12.83 – 37.10 | <0.001 |
| Distance from agricultural land (m) | 0.05 | 0.03 – 0.09 | <0.001 |
| Distance from oil palm plantation (m) | 0.96 | 0.92 – 1.00 | 0.072 |
| Distance from irrigated farmland (m) | 1.34 | 1.25 – 1.43 | <0.001 |
| Distance from old forest (primary) (m) | 0.94 | 0.92 – 0.97 | <0.001 |

**Supplemental Table 11. Adjusted odds ratios for fixed effects for environmental and land-use factors impact the odds of sighting a bat.** The combination of predictors which yielded the model with the highest log likelihood is shown (-19622.72) with household as the random effect. A univariate analysis was first used to select from possible explanatory variables; those with p$\leq$0.2 were included in the development of a final model. This model was made with the lme4 package(1) and analysed with the broom.mixed package(2) in RStudio version 1.3.1093(3, 4).

| **Odds of poultry ownership** | | | |
| --- | --- | --- | --- |
| Household-level risk factors | | | |
| Predictor variable | Adjusted Odds Ratios | 95% CI | P value |
| Normalized differential vegetation index | 1.34 | 1.31 – 1.36 | <0.001 |
| Population density (per km^2^) | 1.03 | 1.01 – 1.05 | 0.008 |
| Elevation (meters above sea level) | 0.22 | 0.17 – 0.30 | <0.001 |
| Slope (degrees) | 0.93 | 0.90 – 0.96 | <0.001 |
| Aspect (degrees) | 1.02 | 1.01 – 1.03 | 0.004 |
| Distance from sea (m) | 1.48 | 1.20 – 1.82 | <0.001 |
| Average temperature, 1970 - 2000 (°C) | 0.08 | 0.06 – 0.12 | <0.001 |
| Median diurnal range, 1970 - 2000 (°C) | 1.13 | 0.93 – 1.37 | 0.217 |
| Precipitation seasonality, 1970 - 2000 (coefficient of variation) | 1.43 | 1.31 – 1.57 | <0.001 |
| Distance from bush forest (secondary) (m) | 1.08 | 1.04 – 1.12 | <0.001 |
| Distance from mangroves (m) | 0.21 | 0.14 – 0.34 | <0.001 |
| Distance from rubber plantation (m) | 0.66 | 0.59 – 0.73 | <0.001 |
| Distance from agricultural land (m) | 2.37 | 1.42 – 3.96 | 0.001 |
| Distance from irrigated farmland (m) | 0.93 | 0.89 – 0.98 | 0.003 |
| Distance from old forest (primary) (m) | 1.02 | 1.00 – 1.05 | 0.040 |

**Supplemental Table 12. Adjusted odds ratios for fixed effects for environmental and land-use factors impact the odds of poultry ownership.** The combination of predictors which yielded the model with the highest log likelihood is shown -31006.85) with household as the random effect. A univariate analysis was first used to select from possible explanatory variables; those with p$\leq$0.2 were included in the development of a final model. This model was made with the lme4 package(1) and analysed with the broom.mixed package(2) in RStudio version 1.3.1093(3, 4).

| **Odds of swine ownership** | | | | | | | |
| --- | --- | --- | --- | --- | --- | --- | --- |
| Household-level risk factors | | | | | | | |
| Predictor variable | | Adjusted Odds Ratios | 95% CI | | | P value | |
| Normalized differential vegetation index | 1.80 | | | 1.66 – 1.94 | <0.001 | |  |
| Population density (per km^2^) | 1.03 | | | 0.85 – 1.13 | 0.686 | |  |
| Elevation (meters above sea level) | 8.83 | | | 3.14 – 24.55 | <0.001 | |  |
| Aspect (degrees) | 1.24 | | | 1.19 – 1.29 | <0.001 | |  |
| Distance from sea (m) | 48.01 | | | 18.99 – 121.02 | <0.001 | |  |
| Average temperature, 1970 - 2000 (°C) | 0.41 | | | 0.11 – 1.50 | 0.181 | |  |
| Median diurnal range, 1970 - 2000 (°C) | 0.15 | | | 0.06 – 0.38 | <0.001 | |  |
| Precipitation of the wettest month, 1970 - 2000 (mm) | 1.23 | | | 1.11 – 1.37 | <0.001 | |  |
| Precipitation seasonality, 1970 - 2000 (coefficient of variation) | 4.43 | | | 2.70 – 7.32 | <0.001 | |  |
| Distance from rubber plantation (m) | 2.11 | | | 1.46 – 3.06 | <0.001 | |  |
| Distance from agricultural land (m) | 0.10 | | | 0.06 – 0.19 | <0.001 | |  |
| Distance from oil palm plantation (m) | 0.11 | | | 0.07 – 0.16 | <0.001 | |  |

**Supplemental Table 13. Adjusted odds ratios for fixed effects for environmental and land-use factors impact the odds of swine ownership.** The combination of predictors which yielded the model with the highest log likelihood is shown (-4131.126) with household as the random effect. A univariate analysis was first used to select from possible explanatory variables; those with p$\leq$0.2 were included in the development of a final model. This model was made with the lme4 package(1) and analysed with the broom.mixed package(2) in RStudio version 1.3.1093(3, 4).

# Moran’s *I* test for residual spatial autocorrelation

|  | NHPs | Bats | Poultry | Swine |
| --- | --- | --- | --- | --- |
| P value | < 2.2*10^-16^ | < 2.2*10^-16^ | < 2.2*10^-16^ | 0.1422 |

**Supplemental Table 14. There is spatial autocorrelation for the residuals of all models except that based on swine contact.** Spatial autocorrelation of the residuals was assessed with Moran’s *I* with p < 0.05 considered statistically significant.

# Fixed effects of geostatistical models

|  | Mean | Standard Deviation |
| --- | --- | --- |
| Normalized differential vegetation index | -0.193 | 29.601 |
| Population density (per km^2^) | 0.058 | 0.014 |
| Elevation (meters above sea level) | -0.022 | 0.012 |
| Aspect (degrees) | -0.373 | 0.249 |
| Distance from sea (m) | -0.010 | 0.007 |
| Average temperature, 1970 - 2000 (°C) | 0.473 | 0.709 |
| Median diurnal range, 1970 - 2000 (°C) | -2.667 | 1.753 |
| Maximum temperature of warmest month, 1970 - 2000 (°C) | -0.817 | 0.660 |
| Minimum temperature of coldest month, 1970 - 2000 (°C) | -0.170 | 0.893 |
| Precipitation of the wettest month, 1970 - 2000 (mm) | 3.544 | 1.377 |
| Precipitation seasonality, 1970 - 2000 (coefficient of variation) | -1.546 | 0.205 |
| Distance from bush forest (secondary) (m) | 0.161 | 0.356 |
| Distance from mangroves (m) | -0.463 | 0.045 |
| Distance from rubber plantation (m) | -0.250 | 0.714 |
| Distance from agricultural land (m) | 0.790 | 0.148 |
| Distance from oil palm plantation (m) | -0.467 | 0.953 |
| Distance from irrigated farmland (m) | 0.218 | 0.081 |
| Distance from old forest (primary) (m) | 0.005 | 0.120 |

**Supplemental Table 15. Mean and standard deviation of fixed effects for geostatistical model of NHP contact.** Spatial patterns of mean posterior estimated probability of animal exposure were modelled using R-INLA(5, 6) and the SPDE method(7) as the spatial effect. The final model has a DIC of 33087.32. The fixed effects were mean-centred and scaled before any analysis was run.

|  | Mean | Standard deviation |
| --- | --- | --- |
| Population density (per km^2^) | -0.121 | 0.022 |
| Elevation (meters above sea level) | 2.064 | 0.414 |
| Slope (degrees) | -0.100 | 0.025 |
| Aspect (degrees) | 0.073 | 0.008 |
| Distance from sea (m) | 1.878 | 1.848 |
| Average temperature, 1970 - 2000 (°C) | -2.451 | 2.315 |
| Median diurnal range, 1970 - 2000 (°C) | 4.569 | 1.202 |
| Maximum temperature of warmest month, 1970 - 2000 (°C) | -1.356 | 1.333 |
| Minimum temperature of coldest month, 1970 - 2000 (°C) | 8.565 | 2.344 |
| Precipitation of the wettest month, 1970 - 2000 (mm) | -2.005 | 0.425 |
| Precipitation seasonality, 1970 - 2000 (coefficient of variation) | 4.914 | 0.826 |
| Distance from bush forest (secondary) (m) | -0.209 | 0.109 |
| Distance from mangroves (m) | -1.167 | 1.717 |
| Distance from agricultural land (m) | 3.586 | 2.024 |
| Distance from oil palm plantation (m) | 0.254 | 0.227 |
| Distance from irrigated farmland (m) | 0.297 | 0.424 |
| Distance from old forest (primary) (m) | 0.389 | 0.337 |

**Supplemental Table 16. Mean and standard deviation of fixed effects for geostatistical model of bat contact.** Spatial patterns of mean posterior estimated probability of animal exposure were modelled using R-INLA(5, 6) and the SPDE method(7) as the spatial effect. The final model has a DIC of 29957.03. The fixed effects were mean-centred and scaled before any analysis was run.

|  | Mean | Standard Deviation |
| --- | --- | --- |
| Normalized differential vegetation index | 0.044 | 0.006 |
| Population density (per km^2^) | 0.006 | 0.005 |
| Elevation (meters above sea level) | -0.012 | 0.015 |
| Slope (degrees) | -0.050 | 0.008 |
| Aspect (degrees) | 0.001 | 0.004 |
| Distance from sea (m) | 0.017 | 0.014 |
| Average temperature, 1970 - 2000 (°C) | -0.014 | 0.015 |
| Median diurnal range, 1970 - 2000 (°C) | 0.008 | 0.014 |
| Precipitation seasonality, 1970 - 2000 (coefficient of variation) | 0.014 | 0.013 |
| Distance from bush forest (secondary) (m) | 0.004 | 0.006 |
| Distance from mangroves (m) | -0.006 | 0.015 |
| Distance from rubber plantation (m) | 0.002 | 0.010 |
| Distance from agricultural land (m) | -0.007 | 0.015 |
| Distance from irrigated farmland (m) | -0.017 | 0.009 |
| Distance from old forest (primary) (m) | 0.003 | 0.007 |

**Supplemental Table 17. Mean and standard deviation of fixed effects for geostatistical model of poultry contact.** Spatial patterns of mean posterior estimated probability of animal exposure were modelled using R-INLA(5, 6) and the SPDE method(7) as the spatial effect. The final model has a DIC of 60218.65. The fixed effects were mean-centred and scaled before any analysis was run.

|  | Mean | Standard Deviation |
| --- | --- | --- |
| Normalized differential vegetation index | 0.588 | 0.039 |
| Population density (per km^2^) | 0.025 | 0.066 |
| Elevation (meters above sea level) | 2.171 | 0.507 |
| Aspect (degrees) | 0.214 | 0.020 |
| Distance from sea (m) | 3.741 | 0.463 |
| Average temperature, 1970 - 2000 (°C) | -0.859 | 0.638 |
| Median diurnal range, 1970 - 2000 (°C) | -1.807 | 0.469 |
| Precipitation of the wettest month, 1970 - 2000 (mm) | 0.206 | 0.053 |
| Precipitation seasonality, 1970 - 2000 (coefficient of variation) | 1.491 | 0.252 |
| Distance from rubber plantation (m) | 0.732 | 0.186 |
| Distance from agricultural land (m) | -2.236 | 0.300 |
| Distance from oil palm plantation (m) | -2.201 | 0.213 |

**Supplemental Table 18. Mean and standard deviation of fixed effects for geostatistical model of swine contact.** Spatial patterns of mean posterior estimated probability of animal exposure were modelled using R-INLA(5, 6) without the spatial effect. The final model has a DIC of 8288.93. The fixed effects were mean-centred and scaled before any analysis was run.

# Standard deviation of geostatistical models


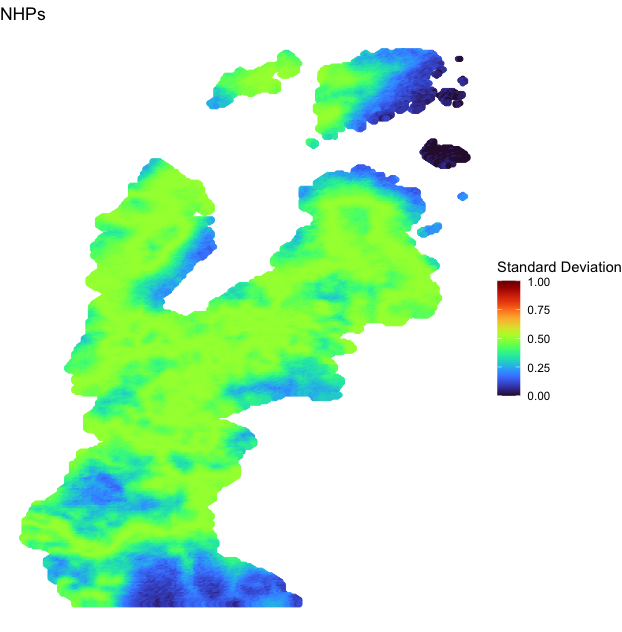

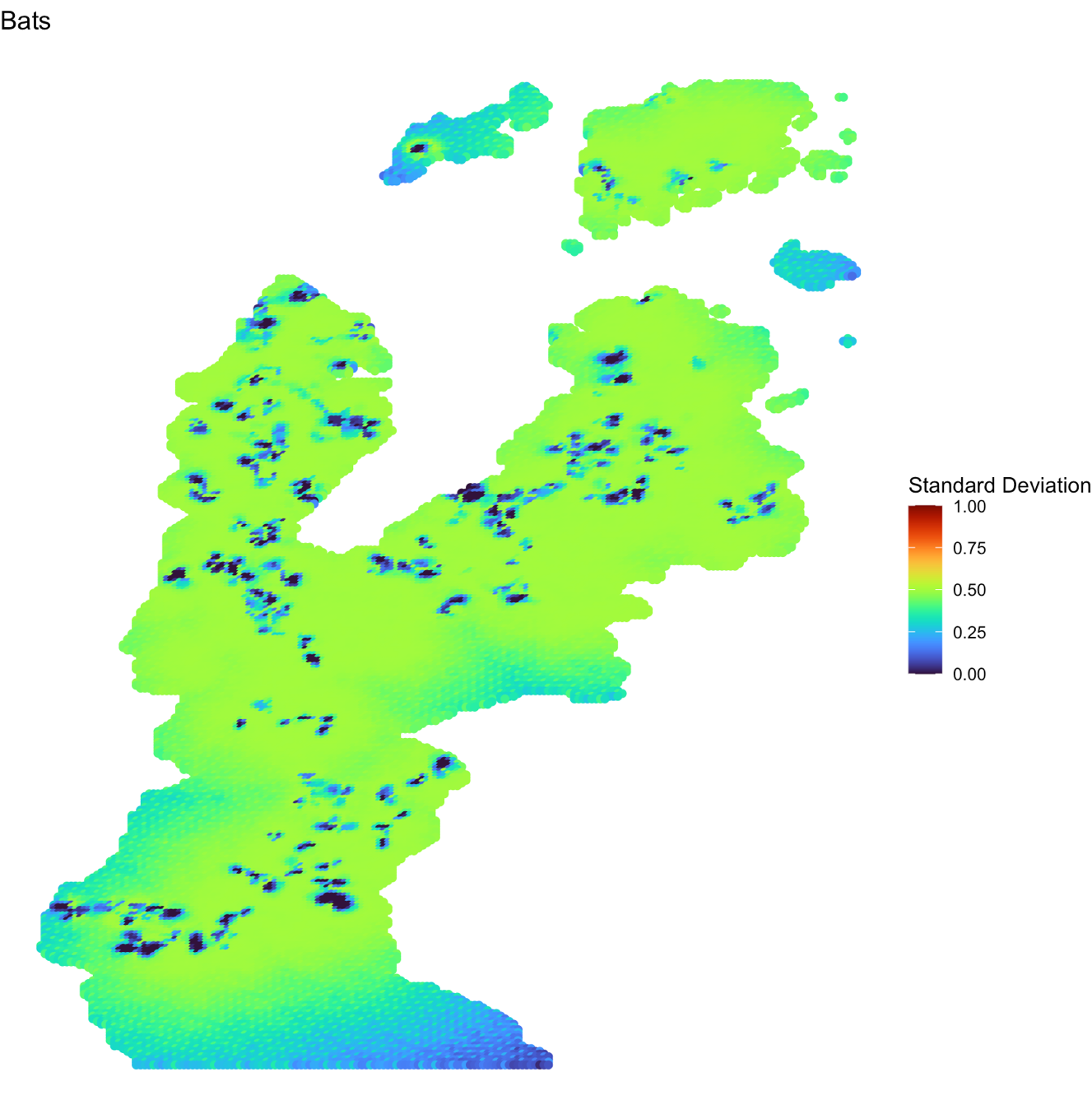
 **
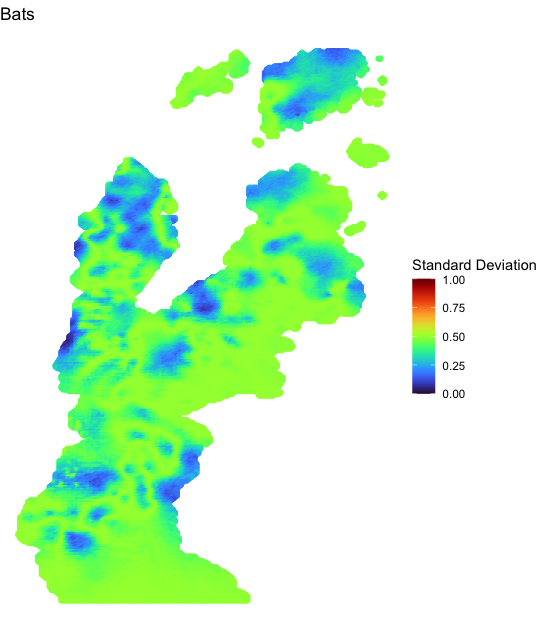
**

**A**

**B**


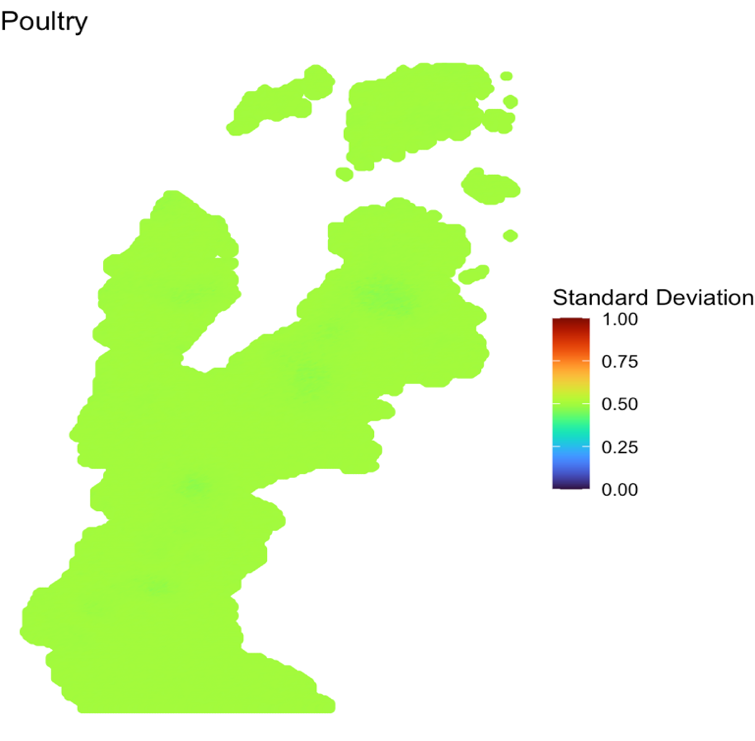

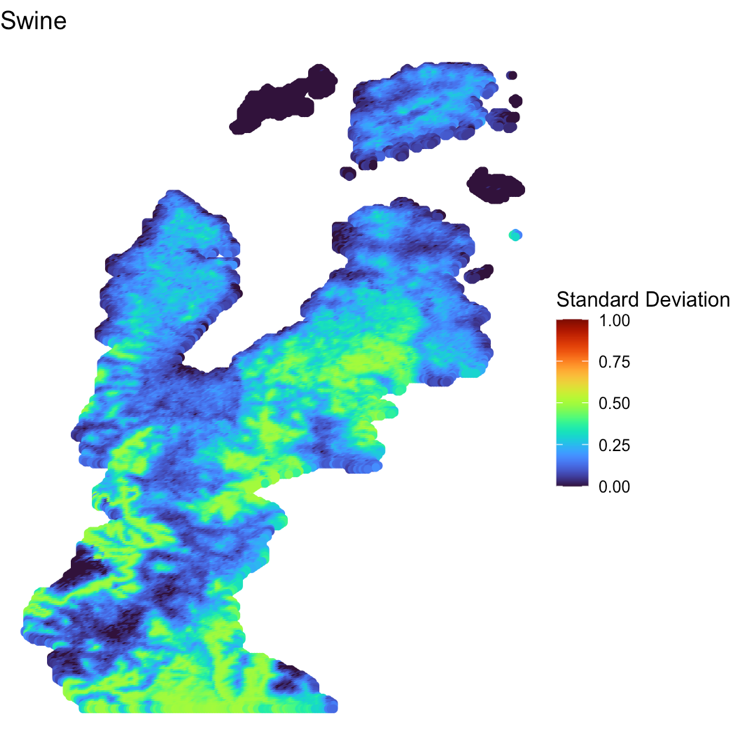


**D**

**C**

**Supplemental Figure 1. Standard deviation of predicted probability of exposure to zoonotic reservoir species.** Spatial patterns of mean posterior estimated probability of animal exposure were modelled using INLA(5, 6) and the SPDE method(7) as the spatial effect. The maps here represent the uncertainty of the predictions shown in Figure 4 where (A) models NHPs, (B) models for bats, (C) models poultry, and (D) models swine. For the model intercepts and fixed effects coefficients, weakly informative priors of Normal (0, 100) were used(8).

# Comparison of geostatistical models with and without spatial effect


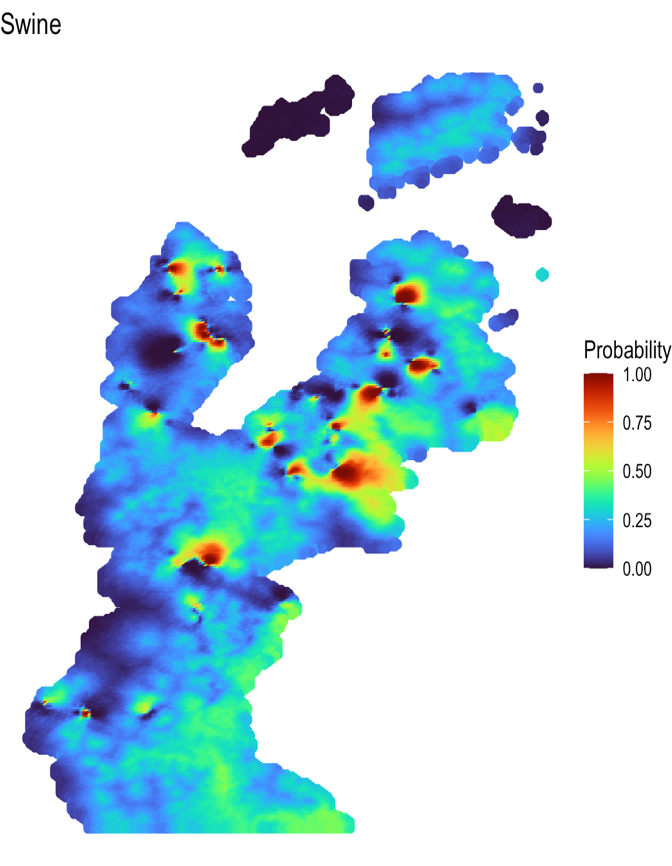

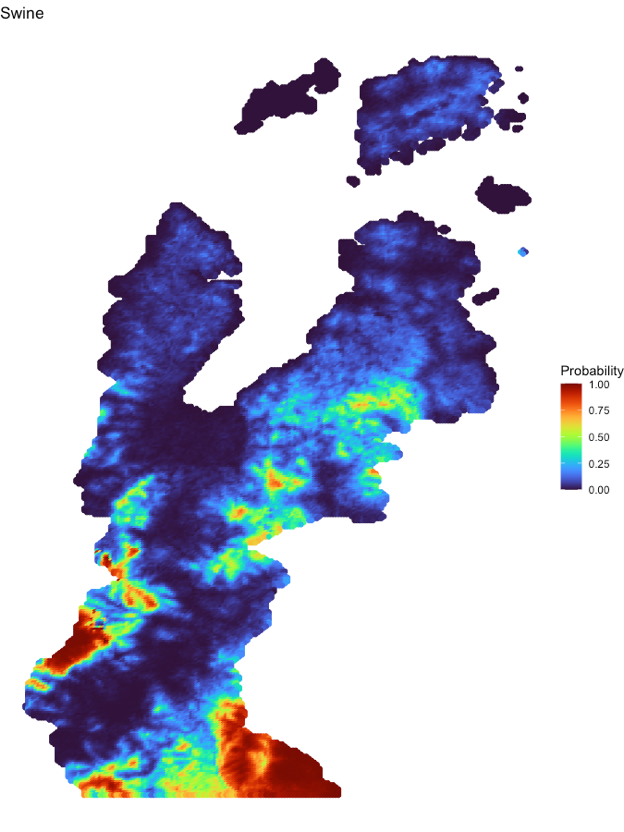


**B**

**A**

**Supplemental** **Figure 2. Model without Matérn covariance function is a better fit for swine exposure.** Deviance information criterions for the model (A) with a spatial effect and (B) without the spatial effect are 20030.48 and 8288.93, respectively.

References

1. Bates D MM, Bolker B, Walker S Fitting Linear Mixed-Effects Models Using Lme4. *Journal of Statistical Software* (2015) 67(1):1-48. doi: 10.18637/jss.v067.i01.

2. Ben Bolker DR, Dieter Menne, Jonah Gabry, Paul Buerkner, Christopher Hua, William Petry, Joshua Wiley, Patrick Kennedy, Eduard Szöcs, Indrajeet Patil, Vincent Arel-Bundock. *Broom.Mixed, Tidying Methods for Mixed Models*. 0.2.7 ed(2021).

3. R Core Team. R: A Language and Environment for Statistical Computing. Vienna, Austria: R Foundation for Statistical Computing (2020) [02 01 2021]. Available from: <https://www.R-project.org/>.

4. RStudio Team. *Rstudio: Integrated Development for R.* Boston, MA: RStudio, PBC (2020).

5. A Breakthrough in the Development of a Universal Flu Vaccine: The University of Oxford [cited 2021 15 03 2021]. Available from: <https://www.ox.ac.uk/research/research-impact/breakthrough-development-universal-flu-vaccine>.

6. Martins TG, Simpson D, Lindgren F, Rue H. Bayesian Computing with Inla: New Features. *Computational Statistics & Data Analysis* (2013) 67:68-83. doi: <https://doi.org/10.1016/j.csda.2013.04.014>.

7. Lindgren F, Rue H, Lindström J. An Explicit Link between Gaussian Fields and Gaussian Markov Random Fields: The Stochastic Partial Differential Equation Approach. *Journal of the Royal Statistical Society: Series B (Statistical Methodology)* (2011) 73(4):423-98. doi: <https://doi.org/10.1111/j.1467-9868.2011.00777.x>.

8. Simpson D, Rue H, Riebler A, Martins TG, Sørbye SH. Penalising Model Component Complexity: A Principled, Practical Approach to Constructing Priors. *Statistical Science* (2017) 32(1):1-28, .
